# Thermal Regulation of CO_2_ Activation Pathways via Interfacial Water Restructuring Enables Ampere-Level, Near-Unity CO Electrosynthesis

**DOI:** 10.1021/jacs.6c02677

**Published:** 2026-05-20

**Authors:** Yang Li, Qixin Yuan, Xiang Lyu, Juan D. Jimenez, Dali Yang, Lu Ma, Xiaoxuan Yang, Jianchun Jiang, Alexey Serov, Mengmeng Fan, Jingjie Wu

**Affiliations:** 1 Department of Chemical and Environmental Engineering, 2514 University of Cincinnati, Cincinnati, Ohio 45221, United States; 2 Jiangsu Co-Innovation Center of Efficient Processing and Utilization of Forest Resources, International Innovation Center for Forest Chemicals and Materials, College of Chemical Engineering, 74584 Nanjing Forestry University, Nanjing 210037, China; 3 Electrification and Energy Infrastructures Division, 6146 Oak Ridge National Laboratory, Oak Ridge, Tennessee 37831, United States; 4 Chemistry Division, 8099 Brookhaven National Laboratory, Upton, New York 11973, United States; 5 National Synchrotron Light Source II, 8099 Brookhaven National Laboratory, Upton, New York 11973, United States

## Abstract

Electrochemical reduction of CO_2_ to CO is a key step in carbon utilization technologies, yet maintaining high CO selectivity under elevated temperatures relevant to industrial membrane-electrode-assembly (MEA) electrolyzers remains challenging due to the competing hydrogen evolution reaction (HER). Additionally, the temperature dependence of CO selectivity on Cu-based catalysts has remained largely unexplored. Here, we demonstrate that incorporating atomic In or Sn into Cu fundamentally reshapes the selectivity of Cu catalysts at elevated temperatures. Dilute alloy catalysts, In_1_Cu and Sn_1_Cu, achieve >95% FE of CO over a broad current-density window (0.1–1.1 A cm^–2^) at 60 °C in MEA electrolyzers, far exceeding their performance at ambient temperature. In situ attenuated total reflection surface-enhanced infrared absorption spectroscopy suggests that elevating temperature depletes interfacial water activity, which favors a shift in CO_2_ activation from a proton-coupled *COOH pathway toward an electron-driven *COO^–^-associated pathway, while also suppressing HER and CO hydrogenation. In contrast, benchmark CO-selective catalysts such as Ag exhibit minimal temperature-induced changes in CO production at 20–60 °C. These findings identify temperature as an unavoidable yet previously underutilized operating parameter in MEA electrolyzers for high-rate, selective CO production on Cu-based catalysts.

## Graphical abstract

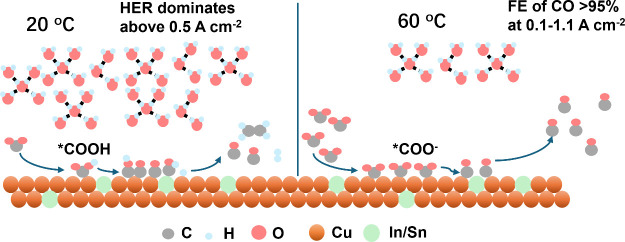

## Introduction

Electrochemical carbon dioxide reduction reaction (CO_2_RR) powered by renewable electricity offers a direct pathway to recycle CO_2_ into fuels and commodity chemicals.^1,2^ For economic deployment, CO_2_ electrolyzers must operate simultaneously at high selectivity toward a single product, high current density, high energy efficiency and high carbon efficiency.^3,4^ Among CO_2_RR products, multicarbon (C_2+_) products such as ethylene (C_2_H_4_) and ethanol (C_2_H_5_OH) are attractive because of their high energy density and market value, but their formation at optimal selectivity requires large overpotentials (>−0.7 V) and a 12-electron transfer, making them energetically costly.^5,6^ In contrast, carbon monoxide (CO) is a more energy-efficient product: it can be generated at overpotentials as low as −0.08 V by efficiently activating CO_2_ and serves as a versatile feedstock for downstream thermochemical upgrading.^7^ Moreover, techno-economic analyses indicate that the most energy-efficient route to C_2_H_4_ may be a cascade strategy, in which CO_2_ is first electrochemically reduced to CO and subsequently converted to C_2_H_4_ in a second step, particularly at elevated operating temperatures, where cell voltages are lower.^8^ Indeed, industrial membrane-electrode-assembly (MEA) cell stacks inevitably operate at higher temperatures (40–70 °C) due to energy inefficiency.^9^ Bridging the gap from laboratory demonstrations to scalable CO_2_-to-CO electrolysis, therefore, requires catalyst materials that maintain performance under heated operating conditions. Yet most CO-selective catalysts reported to date have been evaluated almost exclusively near room temperature.

Cu presents a particularly intriguing but complex catalyst that supports competing C_1_ and C_2+_ pathways. This bifurcation originates from the fate of the key intermediate *CO: it can either desorb as CO or undergo further hydrogenation to *CHO, enabling subsequent C–C coupling (for example via *CO–CHO).^10,11^ Because hydrogen in C_2_H_4_ and C_2_H_5_OH is supplied mainly by interfacial adsorbed hydrogen (*H) rather than direct transfer from interfacial water,^12^ the structure and dynamics of the interfacial water network critically regulate selectivity.^13^ As a result, Cu typically exhibits a characteristic potential-dependent product distribution:^5^ CO tends to dominate at lower overpotentials where *H coverage is low, while increased *H availability at more cathodic overpotentials accelerates *CO hydrogenation, thus favoring C_2+_ formation.^14^ Notably, CO formation is uniquely promoted by a strongly hydrogen-bonded interfacial water network that suppresses *H.^15^ Similar to Cu, our recent study demonstrated that increasing CO_2_ pressure, thereby repelling *H, monotonically enhances CO selectivity on Ag at elevated temperatures (70–80 °C).^16^ Furthermore, 100% CO selectivity has been achieved on Ag in nonaqueous CO_2_ reduction, reinforcing that CO formation does not intrinsically require *H.^17^

One strategy to decrease the *H surface coverage on Cu is alloying Cu with p-block elements such as Sn and In that intrinsically bind *H more weakly than Cu.^18,19^ Dilute alloy catalysts (denoted as Sn_1_Cu or In_1_Cu) have been reported to suppress the hydrogen evolution reaction (HER) and enhance the CO selectivity.^20–25^ However, these materials have also been reported to produce large fractions of C_2+_ products with FE over 50%,^26–28^ especially at more negative potentials where *H becomes prevalent.^29^ This apparent contradiction likely arises from the extreme sensitivity of these alloys to the interfacial microenvironment, which varies widely across these studies depending on electrolyte concentration, ionomer identity, CO_2_ pressure, temperature, potential/current density, cell architecture (e.g., flow cells vs MEA cells) and even electrode fabrication method (e.g., In_1_Cu directly deposited onto the membrane in MEA cell showing FE of C_2_H_4_ of 85%).^28^ For example, concentrated electrolytes (e.g., 1 M versus 0.1 M) disrupt the interfacial hydrogen-bonding network and increase interfacial *H generation,^30^ favoring C_2+_ products formation over CO.^31–33^ Likewise, the choice of ionomer (cation- versus anion-conducting) can reshape the interfacial environment through Donnan exclusion (K^+^ or OH^–^) and modify local CO_2_/H_2_O ratio,^34,35^ which directly regulates *H availability. Despite extensive reports on Sn_1_Cu and In_1_Cu catalysts, a unifying framework linking interfacial water environment, temperature, and product selectivity remains lacking. Critically, the temperature response of such materials remains unexplored, despite its central relevance to industrial operation.

Herein, we demonstrate that temperature provides a powerful and previously unrecognized lever to control selectivity on In_1_Cu and Sn_1_Cu catalysts and exploit the unique temperature-response of these materials to achieve near unity selectivity of CO at ampere-level current density under elevated temperatures. By systematically probing their temperature-dependent behavior, we discover that elevated temperature (e.g., 60 °C) directly repels interfacial H_2_O on In_1_Cu and Sn_1_Cu while simultaneously triggers a *COO^–^ activation pathway, promoting FE of CO to >95% over a wide current density window (100–1,100 mA cm^–2^), far beyond what is achievable at ambient conditions. Notably, this temperature-enabled boost is unique to Cu-based dilute alloy surfaces, while benchmark CO catalysts such as Ag, Zn, and CoPc show minimal gain in CO yield upon heating to 60 °C. Given that practical electrolyzers inevitably operate under heated conditions, our findings reveal a new paradigm for exploiting temperature as a design variable rather than a liability, unlocking industrially relevant, high-rate CO_2_-to-CO conversion.

## Results and Discussion

### Synthesis and Characterization of In_1_Cu and Sn_1_Cu Catalysts

We first synthesized Cu­(OH)_2_ precursors by continuous precipitation of Cu­(NO_3_)_2_ into concentrated NaOH, which yields a fibrous Cu­(OH)_2_ scaffold (Figure S1). Dilute alloy catalysts, In_1_Cu or Sn_1_Cu, were obtained by in situ electrochemical reduction of In or Sn-incorporated Cu­(OH)_2_ nanofibers (denoted as pre-In_1_Cu or pre-Sn_1_Cu) carried out at 60 °C under 300 mA cm^–2^ for 2 h, which preserve the fibrous morphology of Cu­(OH)_2_ (Figures S2 and S3 and Figure 1a). Powder X-ray diffraction (XRD) patterns of pre-In_1_Cu and pre-Sn_1_Cu show peaks indexed to Cu­(OH)_2_ (PDF 04–009–4366) (Figure S4), while In_1_Cu and Sn_1_Cu show metallic Cu peaks, absence of any discernible aggregation of In/Sn metallic or oxide clusters (Figure 1b). Scanning transmission electron microscopy coupled with energy-dispersive X-ray spectroscopy (STEM-EDS) reveals a uniform distribution of In or Sn in the precatalysts (Figures S5 and S6). Aberration-corrected TEM and line-intensity profiles of both In_1_Cu and Sn_1_Cu support the presence of isolated In and Sn (Figure 1c–e). X-ray photoelectron spectroscopy (XPS) was employed to assess the near-surface composition and chemical states of the catalysts. Compared with the precatalysts, the Cu 2p_3/2_ spectra of In_1_Cu and Sn_1_Cu show a pronounced attenuation of Cu­(II)-associated satellite features, indicating a reduction of surface Cu species valence (Figures S7 and S8). Additionally, the In 3d and Sn 3d regions confirm that In and Sn are predominantly present in oxidized environments in the precatalysts, and both exhibit a slight shift to lower binding energy in In_1_Cu and Sn_1_Cu (Figure S9and Figure 1f), indicating increased electron density on In/Sn.^36,37^ Notably, XPS reveals surface atomic fractions of 9.2 at. % In and 13.5 at. % Sn for optimized In_1_Cu and Sn_1_Cu, respectively, whereas inductively coupled plasma mass spectrometry (ICP-MS) quantifies bulk contents of 1.5 at. % In and 4.2 at. % Sn, indicating a pronounced surface enrichment of In/Sn. We further employed X-ray absorption spectroscopy (XAS) to elucidate the local electronic structure and coordination environment of the catalysts. At the Cu K-edge, the XANES of pre-In_1_Cu and pre-Sn_1_Cu lies between the Cu_2_O/CuO references (Figure S10), indicating partially oxidized Cu species. Consistently, the Fourier-transformed Cu K-edge EXAFS displays a dominant first-shell peak at 1.5 Å, characteristic of Cu–O coordination (Figure S11). In contrast, the Cu K-edge XANES of In_1_Cu and Sn_1_Cu closely resembles Cu foil (Figure 1g), accompanied by the emergence of Cu–Cu contribution and a suppressed Cu–O signal in EXAFS (Figure 1h), evidencing a predominant metallic Cu state. Element-specific EXAFS further reveals that In and Sn remain highly dispersed. For precatalysts, the In and Sn K-edge spectra are dominated by In–O and Sn–O coordination, respectively, without discernible In–In or Sn–Sn scattering features (Figures S12 and S13), indicating the absence of metallic In/Sn aggregation. Although Sn_1_Cu retains an oxidized Sn K-edge XANES line shape relative to Sn foil (Figure S14), the Sn K-edge EXAFS of Sn_1_Cu shows the emergence of a Cu–Sn contribution together with a reduced Sn–O amplitude, suggesting doping of atomic Sn into Cu lattice (Figure 1i).

**Figure 1:**
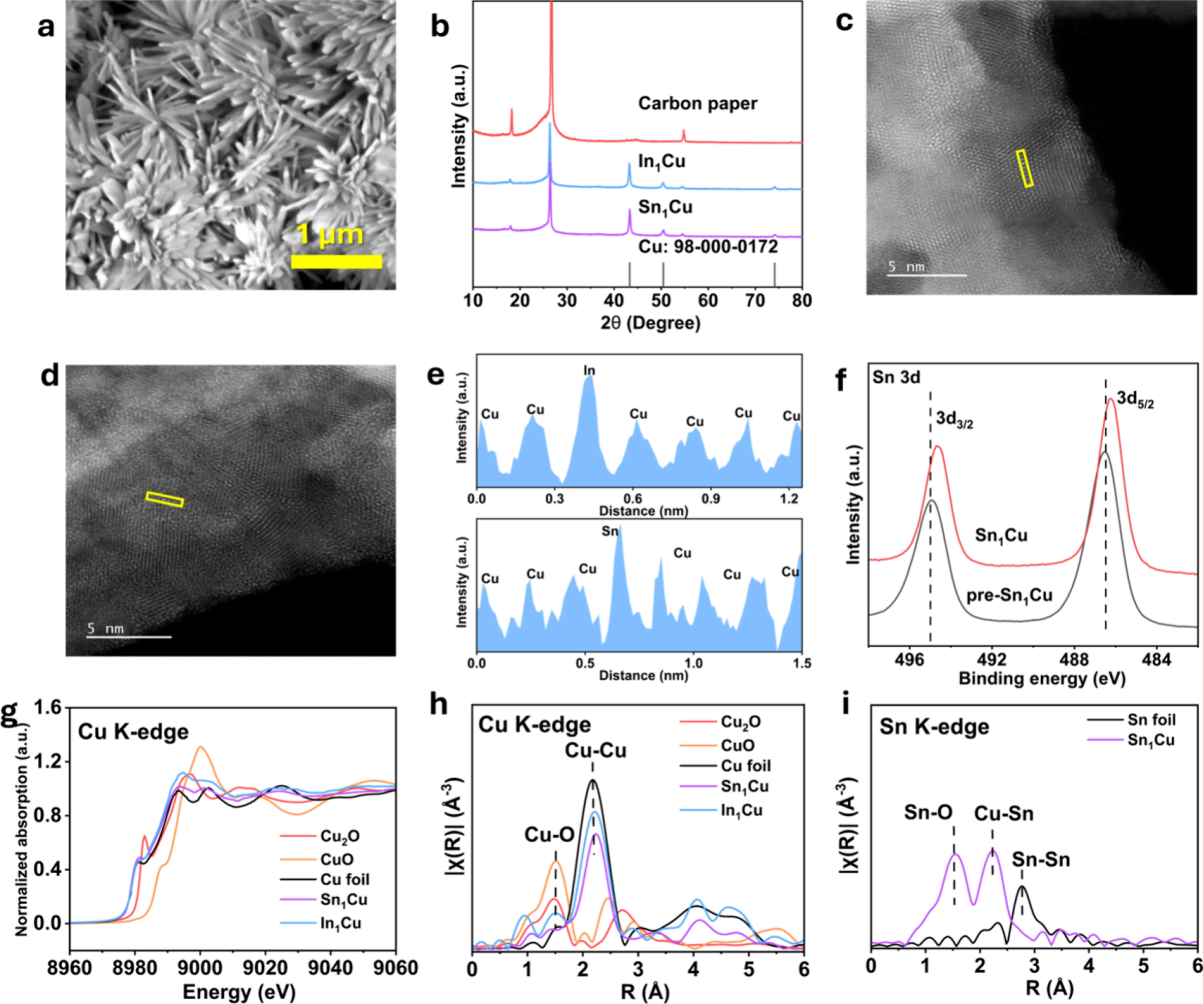
Structural characterizations of In_1_Cu and Sn_1_Cu. (a) SEM image of the Sn_1_Cu catalyst. (b) XRD patterns of Cu, In_1_Cu and Sn_1_Cu catalysts. (c-d) Aberration-corrected TEM of In_1_Cu (c) and Sn_1_Cu (d). (e) Line-intensity profiles of In_1_Cu and Sn_1_Cu as outlined in (c,d). (f) High-resolution Sn 3d XPS spectra of pre-Sn_1_Cu and Sn_1_Cu. (g) Cu K-edge XANES spectra of In_1_Cu and Sn_1_Cu, benchmarked by Cu foil, Cu_2_O and CuO. (h) FT-EXAFS spectra at Cu K-edge of In_1_Cu and Sn_1_Cu, benchmarked by Cu foil, Cu_2_O and CuO. (i) FT-EXAFS spectra at Sn K-edge of Sn_1_Cu, benchmarked by Sn foil. R, apparent radial distance.

### Temperature-Dependent Electrochemical Performance of In_1_Cu and Sn_1_Cu

We evaluated the electrocatalytic CO_2_ reduction performance of In_1_Cu and Sn_1_Cu over a wide temperature range (20–80 °C) in an MEA cell using 0.1 M KHCO_3_ as the anolyte. At 20 °C, In_1_Cu exhibits a typical current-density-dependent product distribution (Figure 2a).^5^ At 100 mA cm^–2^, the relatively low CO_2_ consumption rate together with sluggish H_2_O dissociation rate leads to high *CO_2_ coverage but limited *H availability. Under these conditions, *CO preferentially desorbs, resulting in a dominant CO production (FE of CO = 67%) with minimal H_2_ (FE of H_2_ = 3%). Increasing the current density to 300 mA cm^–2^ accelerates CO_2_ consumption and enhances *H formation, promoting *CO hydrogenation and yielding a total FE of C_2+_ of 42%. The FE of C_2+_ peaks at 45% at 500 mA cm^–2^. At current densities of above 500 mA cm^–2^, rapid H_2_O dissociation combined with CO_2_ mass-transport limitations causes HER to dominate.^14^ Raising the temperature to 40 °C dramatically shifts selectivity toward CO from 100 to 500 mA cm^–2^, with FE of CO exceeding 85% and FE of H_2_ suppressed to 1% (Figure 2b). The maximum FE of C_2+_ decreases to 35% and shifts to 700 mA cm^–2^. HER becomes dominant (FE of H_2_ > 50%) only above 900 mA cm^–2^. This trend, higher FE of CO, suppressed FE of H_2_, and a progressively weakened and shifted C_2+_ window, continues as the temperature further increases to 50 and 60 °C (Figure S15 and Figure 2c). Notably, at 60 °C, FE of CO exceeds 95% even at 1,100 mA cm^–2^ with FE of H_2_ below 4%, whereas the peak FE of C_2+_ is reduced to 12% and shifts to 1,300 mA cm^–2^. At higher temperatures of 70 and 80 °C, FE of H_2_ increases modestly (8%–14%) across 100–900 mA cm^–2^ due to reduced CO_2_ solubility, and C_2+_ products are almost completely suppressed, leaving CO as the dominant product from 100 to 1,500 mA cm^–2^ (Figure S16 andFigure [Fig fig2]d).

**Figure 2:**
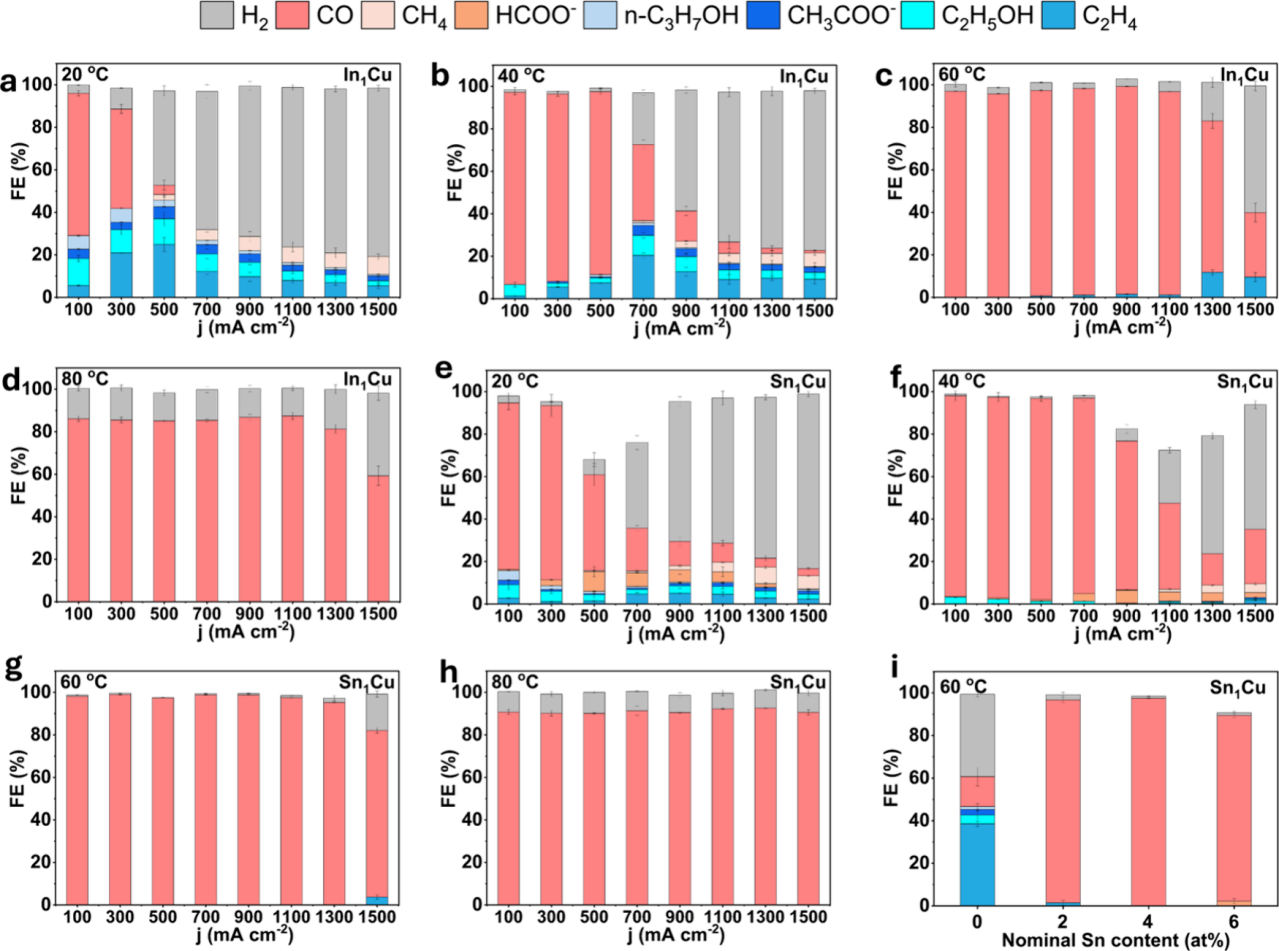
CO_2_ reduction performance of In_1_Cu and Sn_1_Cu under elevated temperatures. (a-d) Product distribution of CO_2_ reduction on In_1_Cu under 20 °C (a), 40 °C (b), 60 °C (c), 80 °C (d). (e-h) Product distribution of CO_2_ reduction on Sn_1_Cu under 20 °C (e), 40 °C (f), 60 °C (g), 80 °C (h). (i) FE for all products on Sn_1_Cu with different nominal Sn contents recorded at 1,100 mA cm^–2^ and 60 °C. The FE values are means, while error bars represent the standard deviation from three independent measurements (*n* = 3).

At 20 °C, Sn_1_Cu exhibits predominant CO production (80% FE of CO) with low FE of C_2+_ (10%) and minimal H_2_ (1%) between 100 and 300 mA cm^–2^ (Figure 2e). At 500 mA cm^–2^, formate appears with a FE of 9%, accompanied by a loss in total FE. Control experiments indicate that this loss may be caused by the anodic oxidation of formate when IrO_2_ is used as the anode catalyst (Figure S17). At current densities above 700 mA cm^–2^, HER becomes dominant. Upon increasing the temperature from 40 to 60 °C, the CO-dominated current-density window significantly widens for Sn_1_Cu, and FE of H_2_ remains suppressed at 1% (Figure 2f,g and Figure S18). Notably, at 60 °C, Sn_1_Cu sustains a FE of CO of 98% over a broad current density range from 100 to 1,100 mA cm^–2^ and retains 95% even at 1,300 mA cm^–2^. With temperatures further elevated to 70 and 80 °C, HER gradually increases within the CO-dominant current-density regime (Figure S19and Figure 2h). Nevertheless, even at 80 °C, Sn_1_Cu still preserves a high FE of CO above 90% across 100 to 1,500 mA cm^–2^, highlighting its exceptional robustness for high-temperature CO production.

For both In_1_Cu and Sn_1_Cu, the full cell voltage decreases monotonically with temperature (Figures S20 and S21). Control experiments with Pt cathodes, which produce only H_2_, show a similar voltage decrease (Figure S22), indicating that the voltage drop with temperature mainly arises from enhanced anode kinetics and reduced ohmic losses.

At 20 °C, a gradual increase in In or Sn content systematically suppresses the formation of C_2+_ products and H_2_, while enhancing the FE of CO up to an optimum (bulk concentration 1.5 at. % for In and 4.2 at. % for Sn), beyond which the FE of formate rises (Figures S23 and S24). At 60 °C, low dopant levels yield high FE of CO, highlighting a temperature-enabled amplification of the dopant effect (Figure 2i and Figure S25). Importantly, at 60 °C, even heavy doping of Sn (6 at. %) still maintains the predominance of CO production, while the competing evolution of formate remains negligible.

To summarize, both catalysts exhibit a monotonic increase in peak FE of CO and CO partial current density (j_CO_) with temperature up to 60 °C. Specifically, for In_1_Cu, the peak j_CO_ rises from 140 mA cm^–2^ at 20 °C to 1,052 mA cm^–2^ at 60 °C (Figure 3a), while for Sn_1_Cu it increases from 246 to 1,237 mA cm^–2^ (Figure 3b). In contrast, the peak FE of C_2+_ decreases continuously with temperature (Figure 3c). Slight pressurization to 3 bar effectively suppresses HER for both catalysts at 80 °C, allowing In_1_Cu and Sn_1_Cu to approach unity FE of CO at the ampere-level current density (Figure S26and Figure 3d). The full cell voltage drops slightly as the operating pressure is elevated to 3 bar.

**Figure 3:**
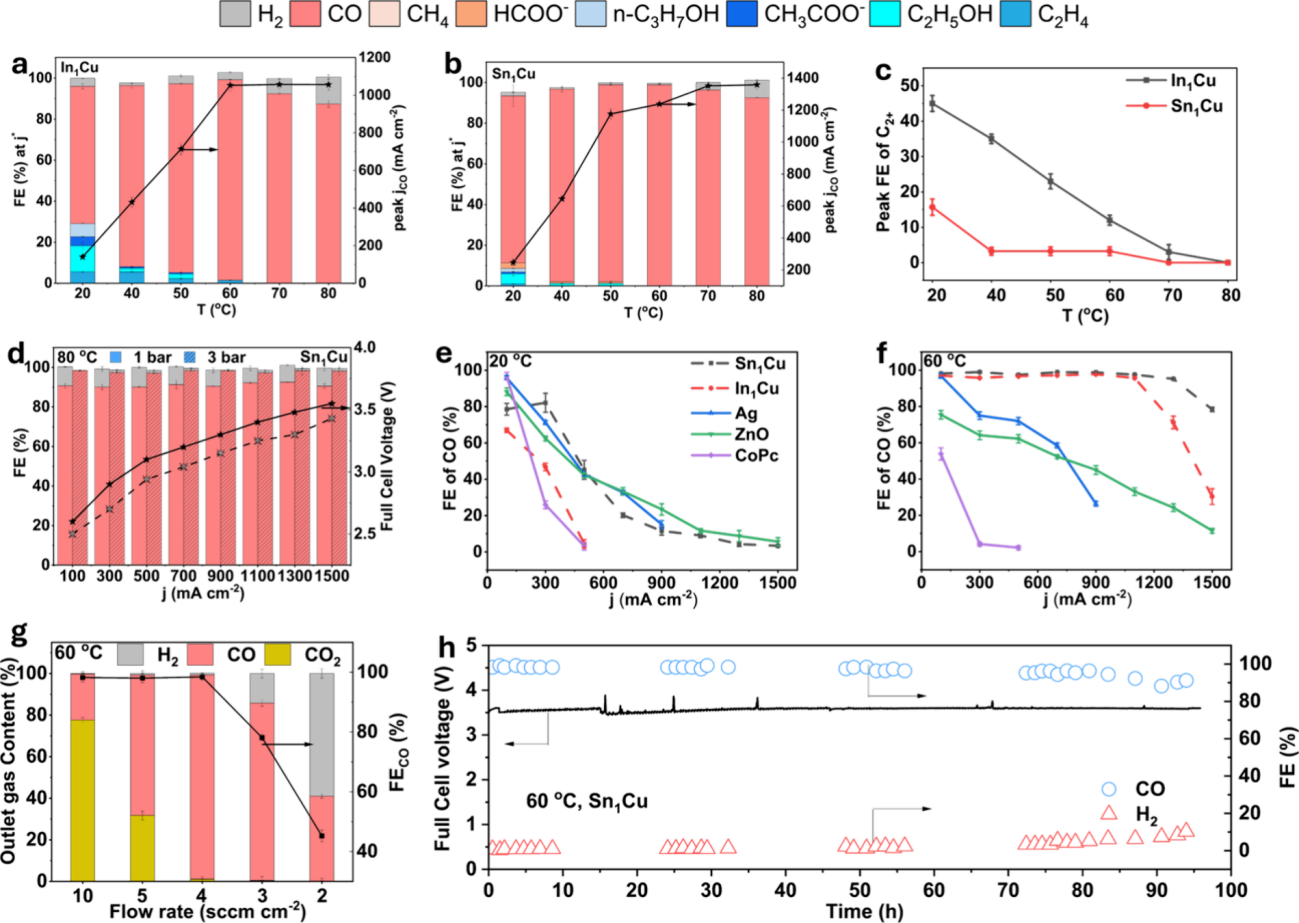
Temperature effect on CO_2_ reduction performance of In_1_Cu and Sn_1_Cu. (a,b) Product distribution on In_1_Cu (a) and Sn_1_Cu (b) at j* under different temperatures, j* means the current density where FE of CO peaks. (c) Peak FE of C_2+_ for In_1_Cu and Sn_1_Cu at different temperatures. (d) Product distribution on Sn_1_Cu under different current densities at 80 °C under 1 and 3 bar. (e,f) FE of CO of Sn_1_Cu, In_1_Cu, and benchmark catalysts Ag, ZnO, and CoPc at 20 °C (e) and 60 °C (f). (g) Outlet gas concentration and FE of CO for Sn_1_Cu at 300 mA cm^–2^ under different CO_2_ flow rates. (h) Stability of Sn_1_Cu operated at 500 mA cm^–2^ under 60 °C (a 40 μm AEM was employed for stability testing). Error bars represent the standard deviation from three independent measurements (*n* = 3).

To benchmark the temperature-enhanced CO-producing capability of In_1_Cu and Sn_1_Cu, we compared their performance with conventional CO-selective catalysts. For a fair comparison, all benchmark cathodes (In_1_Cu, Sn_1_Cu, Ag, ZnO, and CoPc) were prepared using the same electrode-fabrication protocol with the same catalyst loading (0.8 mg cm^–2^) and evaluated in the same MEA cell configuration under identical operating conditions. Classical CO-selective catalysts such as Ag, ZnO, and CoPc exhibit a FE of CO > 90% at 100 mA cm^–2^, clearly outperforming In_1_Cu (66%) and Sn_1_Cu (78%) under ambient conditions (Figure 3e). However, at 60 °C and 1 bar, In_1_Cu and Sn_1_Cu uniquely gain temperature-enhanced CO selectivity at the ampere-level current density, whereas Ag, ZnO, and CoPc show minimal improvement or even performance loss (Figure 3f). These temperature-dependent selectivity trends persist under matched cell-voltage conditions (2.9–3.9 V), with In_1_Cu and Sn_1_Cu exhibiting a more pronounced shift toward CO than Ag upon heating, consistent with the galvanostatic results (Figure S27). We modulated the flow rate of CO_2_ to adjust the conversion. At 60 °C and 1 bar, lowering the CO_2_ flow rate to 4 sccm cm^–2^ still maintains FE of CO of 98% at 300 mA cm^–2^ for Sn_1_Cu, yielding an outlet stream of 98 vol % CO purity (Figure 3g). Further decreasing the flow rate leads to HER owing to the limited CO_2_ flux (Figure S28). Under galvanostatic operation at 500 mA cm^–2^, Sn_1_Cu maintained a FE of CO of 95% for 80 h at 60 °C and 1 bar (Figure 3h). At a higher current density of 1,000 mA cm^–2^, Sn_1_Cu sustained a FE of CO of 90% for 10 h (Figure S29). To exclude catalyst degradation or dissolution, we performed STEM-EDS, XRD, and XPS analyses on the postelectrolysis catalysts, together with ICP-MS analysis of the electrolyte after 95 h stability testing (Figure S30 and Table S1). These results showed no evidence of dopant aggregation, phase segregation, and severe metal leaching. Notably, a 40 μm membrane was used in these stability tests, whereas the performance measurements were conducted with a 20 μm membrane. This modification was necessary because the 20 μm membrane did not provide sufficient long-term stability: after 30 h of operation, the cathode outlet flow rate decreased by 50%, and gas bubbles were observed on the anode side when the electrolysis was paused, indicating increased membrane permeability under prolonged operation.

### Mechanistic Origin for Temperature-Enhanced CO Formation on In_1_Cu and Sn_1_Cu

To elucidate how elevated temperatures promote CO formation on In_1_Cu and Sn_1_Cu, we probed interfacial reaction intermediates using in situ attenuated total reflection surface-enhanced infrared absorption spectroscopy (ATR-SEIRAS) over a potential range from −0.1 to −1.5 V vs RHE at 20 and 60 °C. At 20 °C, both In_1_Cu and Sn_1_Cu exhibit a linearly adsorbed *CO band at 2065 cm^–1^, accompanied by the H–OH bending mode of interfacial water at 1646 cm^–1^ and a *COOH stretching feature at 1380 cm^–1^ (Figure 4a,b). The onset potential of *CO is found to be around −0.10 V vs RHE (Figure S31) and the *CO band intensity decreases slightly with increasingly negative potential, whereas the H_2_O band becomes progressively stronger, indicating accelerated H_2_O dissociation and *CO hydrogenation. The *CO signal is slightly stronger on In_1_Cu than on Sn_1_Cu, indicating a higher surface *CO coverage and a greater tendency toward C–C coupling, consistent with the C_2+_ selectivity. To probe the interfacial hydrogen-bonding environment, we analyzed the O–H stretching region (3000–3800 cm^–1^) of the in situ ATR-SEIRAS spectra. Gaussian deconvolution resolves the broad band into three components centered at 3552, 3417, and 3230 cm^–1^, which are assigned to K^+^-hydrated water (K–H_2_O), moderately hydrogen-bonded water (2-HB H_2_O), and strongly hydrogen-bonded water (4-HB H_2_O), respectively. Stronger hydrogen bonding reflects a more rigid water network that suppresses H_2_O dissociation and limits *H generation at the interface, and the structure of interfacial water is highly correlated with the electrode material.^30,38–41^ Both In_1_Cu and Sn_1_Cu are dominated by 2-HB H_2_O and 4-HB H_2_O, with no discernible contribution from K–H_2_O (Figures S32–35). At −1.10 V vs RHE, In_1_Cu contains 42% 4-HB H_2_O, whereas Sn_1_Cu exhibits a much higher fraction of 75%, suggesting a stronger hydrogen-bonding character of the interfacial water environment (Figure 4c,d). In contrast, bare Cu shows only 34% 4-HB H_2_O and a measurable K–H_2_O population (9%), consistent with a weaker hydrogen-bonded interfacial water environment that may facilitate H_2_O dissociation.

**Figure 4:**
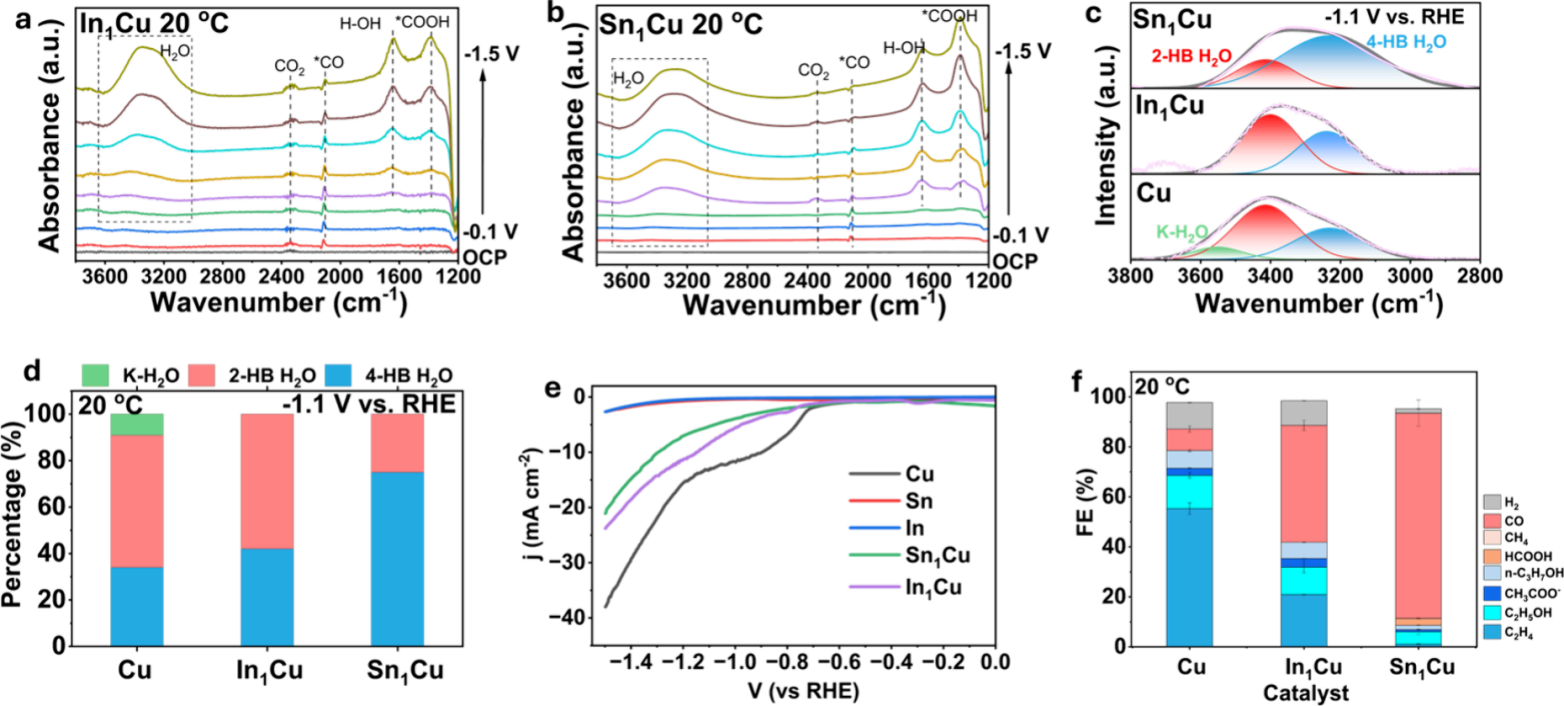
Mechanistic study of CO_2_ reduction on In_1_Cu and Sn_1_Cu at 20 °C. (a,b) In situ ATR-SEIRAS spectra of In_1_Cu (a) and Sn_1_Cu (b) from −0.1 to −1.5 V vs RHE. (c) Gaussian deconvolution of the O–H stretching band of in situ ATR-SEIRAS spectra of Cu, In_1_Cu and Sn_1_Cu at −1.1 V vs RHE. (d) Population of K–H_2_O, 2-HB H_2_O, and 4-HB H_2_O of Cu, In_1_Cu and Sn_1_Cu at −1.1 V vs RHE. (e) LSV curve of Cu, In, Sn, In_1_Cu, and Sn_1_Cu in Ar purged 0.1 M KHCO_3_. (f) Product distribution of Cu, In_1_Cu, and Sn_1_Cu at 300 mA cm^–2^.

These spectroscopic trends correlate directly with HER kinetics (Figure 4e): at 20 °C, Sn_1_Cu exhibits the slowest HER kinetics, followed by In_1_Cu and Cu, which originates from the inert H_2_O dissociation ability of In and Sn. The weaker H_2_O dissociation on Sn_1_Cu compared with In_1_Cu is likely due to its higher dopant concentration (4.2 at. % Sn vs 1.5 at. % In). The ability to split H_2_O and generate *H directly governs the competition between CO and C_2+_ pathways. For example, at 300 mA cm^–2^, bare Cu favors C_2+_ products with a FE of 78%, whereas In_1_Cu and Sn_1_Cu produce progressively less C_2+_ products with FEs of 42% and 10%, respectively (Figure 4f). Conversely, CO selectivity follows the opposite trend, with FE of CO of 8% on Cu, 42% on In_1_Cu, and 82% on Sn_1_Cu (Figure 4f), indicating that incorporating H_2_O-dissociation-inert dopants (In and Sn) into Cu effectively suppresses *H availability and shifts selectivity from C–C coupling toward CO desorption. This trend remains evident at both 20 and 60 °C and under matched cell-voltage conditions (Figures S36 and 37).

At 60 °C, the interfacial ATR-SEIRAS spectra of both In_1_Cu and Sn_1_Cu are dominated by intense *COO^–^ features (Figure 5a,b). The *COO^–^ features located at 1432 and 1298 cm^–1^ are assigned to the asymmetric and symmetric stretching modes, respectively.^42–45^ To validate the assignment of *COO^–^ band, we performed a series of control experiments. Upon replacing ^12^CO_2_ with ^13^CO_2_, both bands exhibit clear redshifts from 1432 to 1370 cm^–1^ and 1298 to 1244 cm^–1^ (Figure S38), confirming their origin from CO_2_-derived surface intermediates rather than temperature-dependent background signals or unrelated interfacial species. Meanwhile, ^12^CO_2_ electrolysis in D_2_O leaves the 1432 and 1298 cm^–1^ bands essentially unchanged, whereas the *COOH band shifts from 1380 to 1353 cm^–1^, consistent with the isotopic sensitivity expected for a protonated carboxyl intermediate but not for *COO^–^ (Figure S39). We further excluded the assignment to carbonate/(bi)­carbonate species. In Ar-purged 1 M K_2_CO_3_ at 60 °C, where carbonate is deliberately abundant, only a distinct carbonate band at 1454 cm^–1^ is observed, clearly different from the 1432 cm^–1^ feature. Moreover, under CO_2_RR conditions on strong acid (pH = 1) environment at 60 °C, where carbonate/(bi)­carbonate species are thermodynamically suppressed and should not accumulate appreciably at the interface, the same pair of bands at 1432 and 1298 cm^–1^ remains clearly visible (Figure S40). Together, these complementary controls support assignment of the 1432 and 1298 cm^–1^ bands to surface-bound *COO^–^ intermediates. In addition, the *CO band weakens relative to 20 °C (Figure S41), aligning with the enhanced CO desorption. Moreover, the H–OH bending mode at 1646 cm^–1^ and the O–H stretching bands (3000–3800 cm^–1^) of interfacial water are strongly attenuated (Figure S42), indicating a lower interfacial water activity as temperature increases. In addition, In_1_Cu exhibits a distinct feature near 3680 cm^–1^ at 60 °C (Figure 5a), which is assigned to noncoordinated interfacial water. This band has been associated with interfacial hydrophobicity or markedly weakened hydrogen-bond connectivity, reinforcing suppressed interfacial water activity on In_1_Cu at 60 °C.^33,46,47^

**Figure 5:**
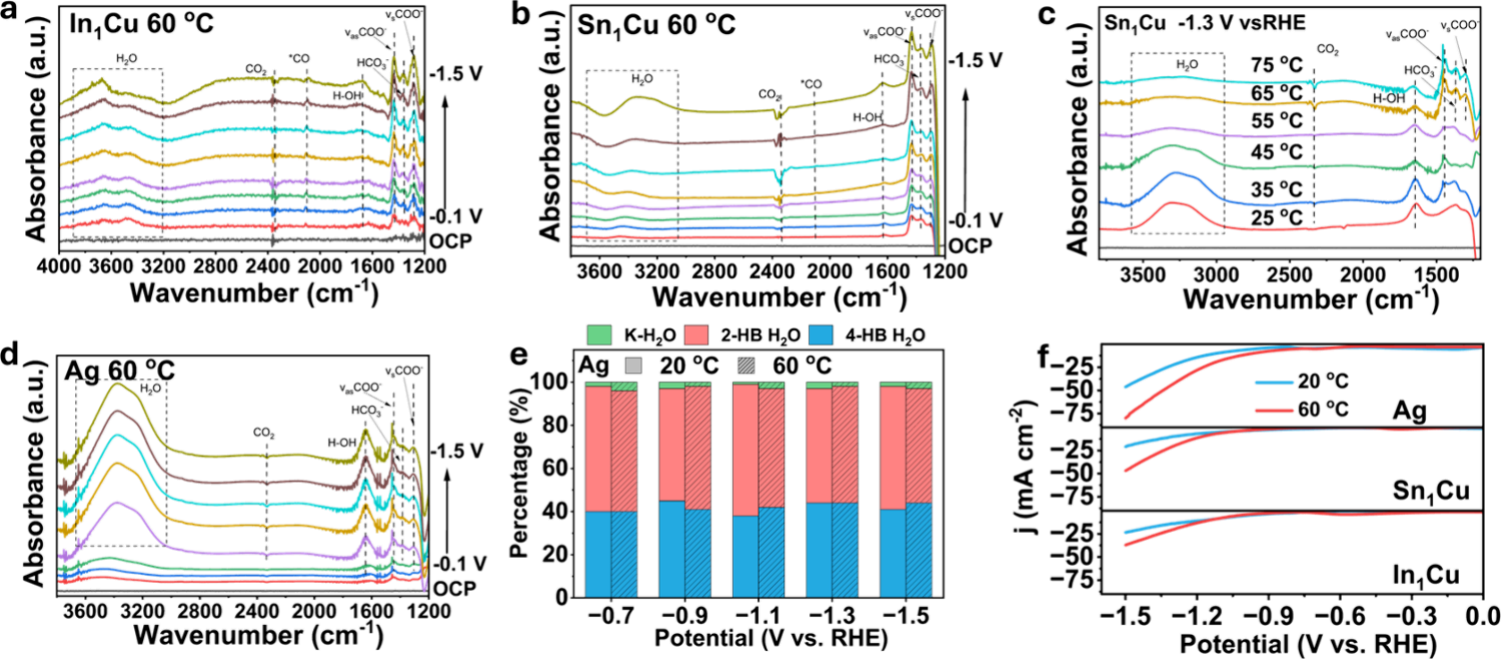
Mechanistic study of CO_2_ reduction on In_1_Cu, Sn_1_Cu, and Ag at 60 °C. (a,b) In situ ATR-SEIRAS spectra of In_1_Cu (a) and Sn_1_Cu (b) from −0.1 to −1.5 V vs RHE at 60 °C. (c) In situ ATR-SEIRAS spectra of Sn_1_Cu at −1.3 V vs RHE from 25 to 75 °C. (d) In situ ATR-SEIRAS spectra of Ag from −0.1 to −1.5 V vs RHE at 60 °C. (e) Comparison of population of K–H_2_O, 2-HB H_2_O, and 4-HB H_2_O for Ag between 20 and 60 °C from −0.7 to −1.1 V vs RHE. (f) LSV curve of In_1_Cu, Sn_1_Cu, and Ag in Ar-purged 0.1 M KHCO_3_ at 20 and 60 °C.

Prior studies established that CO_2_ activation proceeds via either a concerted proton–electron transfer (CPET) pathway (eqs 1 and 4) or a sequential proton–electron transfer (SPET) pathway (eqs 2–4):^48–51^

$$*{\mathrm{CO}}_{2}+H^{+}+e^{-}↔*\mathrm{COOH}$$ 1

$$*{\mathrm{CO}}_{2}+e^{-}↔*{\mathrm{COO}}^{-}$$ 2

$$*{\mathrm{COO}}^{-}+H_{2}O↔*\mathrm{COOH}+{\mathrm{OH}}^{-}$$ 3

$$*\mathrm{COOH}+H^{+}+e^{-}↔*\mathrm{CO}+H_{2}O$$ 4

At 20 °C, In_1_Cu and Sn_1_Cu form *COOH at relatively negative potentials (−0.7 V vs RHE) where H_2_O dissociation becomes favorable, consistent with CPET CO_2_ activation. In contrast, at 60 °C, the emergence of strong *COO^–^ bands at more positive potentials (−0.1 V vs RHE) and their persistence across the measured potential window suggest a greater contribution from a *COO^–^-associated SPET route (Figure 5a,b). Simultaneously, a negative CO_2_ band at 2340 cm^–1^ appears, indicating net CO_2_ consumption at the interface. At 20 °C this band is positive because the CO_2_ feed exceeds consumption, further highlighting faster CO_2_ activation kinetics at 60 °C.

Temperature-resolved in situ ATR-SEIRAS measurements further reveal that the interfacial water activity decreases, *COO^–^ intermediate coverage rises, and CO_2_ consumption rate increases with temperature (Figure 5c). Notably, across the entire temperature range from 20 to 75 °C, even as overall interfacial water activity decreases, strongly hydrogen-bonded water (4-HB H_2_O) remains dominant (Figures S43 and 44). These results suggest that increasing temperature primarily lowers the interfacial water population, while any change in hydrogen-bonding order is less pronounced.

### Why Temperature Uniquely Boosts Cu-Based Dilute Alloys

CO formation is fundamentally limited by CO_2_ adsorption and activation, regardless of whether proton transfer is coupled or not.^17,52–54^ However, substantial efforts have targeted on shifting the CO_2_ activation step from proton-coupled *COOH pathway (eq 1) to the electron-transfer-driven *COO^–^ pathway (eq 2) through materials and electrolyte engineering, which can substantially lower the overpotential of CO formation and accelerating reaction rates.^7,55,56^ For example, ionic liquid electrolytes reduce the free energy of *COO^–^ formation via complexation, leading to a 0.6 V decrease of the CO formation overpotential.^57^ Efficient activation of CO_2_ to *COO^–^ directly correlates with CO formation.^58^ Metals such as Ag, Au, and Cu, with high d-electron availability, can facilitate π back-donation into CO_2_ antibonding orbitals, promoting *COO^–^ formation.^59^ In CoPc, *COO^–^ could be formed when the cobalt center of the complex is in the Co^I^ oxidation states, which enables effective electron donation into the CO_2_ π* antibonding orbital.^49^ Once CO_2_ is activated, a cationic or positively polarized double layer stabilizes the negatively charged *COO^–^ intermediate. In most aqueous systems, solvated alkali-metal cations provide this stabilization through short-range electrostatic interactions and polarization of the adsorbed anion.^60,61^ In addition, positively charged molecular catalyst (e.g., CoPc) could partially substitute for alkali-metal cations, providing weak stabilization of *COO^–^ even in alkali cation free electrolytes.^62^ On the other hand, 100% CO selectivity was achieved in nonaqueous CO_2_ reduction, reinforcing that CO formation does not intrinsically require H_2_O environment,^17^ but is instead governed primarily by the efficiency of CO_2_ activation.

The key mechanistic role of temperature on In_1_Cu and Sn_1_Cu is not to enhance proton transfer, but to suppress interfacial water activity while shifting CO_2_ activation decisively toward the SPET pathway in which *COO^–^ intermediate becomes kinetically accessible. The stabilization of the *COO^–^ is achieved primarily through the electric polarization of remaining interfacial K^+^ under elevated temperatures:^63^ replacing the 0.1 M KHCO_3_ anolyte with pure water collapses CO selectivity and yields over 90% FE of H_2_, accompanied by a marked increase in full cell voltage (5.1 V) at 500 mA cm^–2^. By contrast, operation with 0.1 M KHCO_3_ sustains near-unity (98%) FE of CO at a substantially lower full cell voltage (3.2 V) at 500 mA cm^–2^ (Figure S45).

In contrast, Ag displays nearly identical interfacial water activity and structure, and CO_2_-derived intermediates at 20 and 60 °C (Figure 5d,e and Figures S46–48). As a result, its FE of CO gains little with temperature (Figure S49a). Sn_1_Cu shows a dramatic enhancement in FE of CO accompanied by a 5-fold increase in j_CO_ at 60 °C (Figure 3b).

Although both Ag and Sn_1_Cu favor CO_2_ activation via the *COO^–^ intermediate at 60 °C, their selectivity at the high current region diverges sharply. For Ag, CO selectivity is high at a low current density (FE of CO = 96% at 100 mA cm^–2^), but drops rapidly as the current density increases (74% at 300 mA cm^–2^, and 26% at 900 mA cm^–2^). In contrast, Sn_1_Cu maintains near-unity CO production even at ampere-level operation, for example, FE of CO = 95% at 1,300 mA cm^–2^. The CO_2_ availability at 60 °C is comparable to 20 °C as reduced solubility is offset by increased diffusivity and only minor changes in GDE wetting.^16^ Instead, this divergence is consistent with fundamentally different responses of the interfacial water environment with temperature on these catalysts. Compared with Ag, In_1_Cu and Sn_1_Cu exhibit substantially weaker enhancement of HER as the temperature is increased from 20 to 60 °C (Figure 5f). That is because In_1_Cu and Sn_1_Cu intrinsically suppress interfacial water activity as temperature increases, enabling sustained CO production even under ambient pressure. When the effective interfacial water activity is reduced, such as under elevated CO_2_ pressure (3 bar), Ag similarly recovers high FE of CO to 95% at 1,000 mA cm^–2^ at 60 °C (Figure S49b).^16^ Conversely, using concentrated K^+^ electrolytes could disrupt the interfacial hydrogen-bonding network and increase free H_2_O activity, promoting *H-assisted C_2+_ and formate pathways.^30,33,64,65^ We compare these two interfacial perturbations (temperature and concentrated cation effects) for In_1_Cu and Sn_1_Cu using more concentrated KHCO_3_ anolytes (0.5 and 1.0 M). Increasing K^+^ concentration indeed shifts selectivity away from CO, promoting C_2+_ formation on In_1_Cu while formate formation on Sn_1_Cu (Figure S50). Importantly, however, even under these conditions, raising the temperature to 60 °C shifts the selectivity back toward CO. Both variables therefore reshape the water activity, and under highly concentrated K^+^ conditions (1 M), the cation effect can become more dominant and partially compensate for the temperature effect. Nevertheless, we emphasize that temperature is an independent and important lever for tuning selectivity, particularly under the practical low electrolyte concentration conditions. Highly concentrated anolytes are not preferred because they accelerate salt precipitation during long-term operation in the MEA cell. These observations identify suppression of interfacial water activity as a critical design principle for sustaining high CO selectivity on both Ag and In_1_Cu/Sn_1_Cu at ampere-level current densities, which is controlled by temperature, cation concentration, potentials, and materials.

## Conclusion

This work demonstrates that elevated temperatures fundamentally enhance CO selectivity on H_2_O-disscoiation-inert elements (e.g., In, Sn) modified Cu-based materials. Temperature weakens interfacial water activity, thereby suppressing *CO hydrogenation and the competing HER. In situ ATR-SEIRAS supports a shift from a *COOH-associated proton-coupled activation step at 20 °C toward a water-deficient, *COO^–^-dominated activation pathway at 60 °C. The accelerated CO_2_ activation combined with the suppressed H_2_O availability uniquely promotes high CO productivity for In_1_Cu and Sn_1_Cu, with both catalysts sustaining near-unity FE of CO at 60 °C from 0.1 to 1.1 A cm^–2^, far beyond their performance at ambient temperature. At even higher temperature (>70 °C), modest pressurization further suppresses HER and preserves CO selectivity approaching unity. Given that practical MEA cell stacks intrinsically operate under heated conditions, our findings provide both a mechanistic framework and a directly deployable operating strategy for achieving robust, high-rate CO production on Cu-based catalysts in scalable CO_2_ electrolyzers.

## Supplementary Material



## Notes

Catalyst preparation method; characterizations; electrochemical measurements (PDF)
