# Supplementary material for: Thermal Regulation of CO2 Activation Pathways via Interfacial Water Restructuring Enables Ampere-Level, Near-Unity CO Electrosynthesis
Source: J Am Chem Soc. 2026 May 20;148(21):21807–16. doi: 10.1021/jacs.6c02677 (PMC13244455; doi:10.1021/jacs.6c02677)
Supplement: Supplementary file 1 [file ja6c02677_si_001.pdf]

Supporting Information

Thermal Regulation of CO_2 Activation Pathways via Interfacial Water Restructuring Enables Ampere-Level, Near-Unity CO Electrosynthesis

Yang Li[1,#], Qixin Yuan[2,#], Xiang Lyu[3,#], Juan D. Jimenez[4], Dali Yang[5], Lu Ma[5], Xiaoxuan Yang[3], Jianchun Jiang[2], Alexey Serov[3,], Mengmeng Fan[2,*], Jingjie Wu[1,*]*

[1] Department of Chemical and Environmental Engineering, University of Cincinnati, Cincinnati, OH 45221, USA

[2] Jiangsu Co-Innovation Center of Efficient Processing and Utilization of Forest Resources, International Innovation Center for Forest Chemicals and Materials, College of Chemical Engineering, Nanjing Forestry University, Nanjing, 210037, China

[3] Electrification and Energy Infrastructures Division, Oak Ridge National Laboratory, Oak Ridge, TN 37831, USA

[4] Chemistry Division, Brookhaven National Laboratory, Upton, NY 11973, United States

[5] National Synchrotron Light Source II, Brookhaven National Laboratory, Upton, NY 11973, United States

Y.L., Q.Y. and X.L. contributed equally to this work.

Methods

Chemicals

All chemicals were used without further purification. Copper nitrate trihydrate ($Cu(NO_3)_2 \cdot 3H_2O$, 99%), Potassium bicarbonate ($KHCO_3$, 99.7%) were purchased from Sigma-Aldrich. Sodium hydroxide (NaOH, 95%) was purchased from Fisher. Cobalt phthalocyanine (CoPc) powder, silver nanopowder (20-40 nm) were purchased from Thermo-scientific. Tin chloride ($SnCl_2$, anhydrous) was purchased from Alfa Aesar. Indium nitrate hydrate ($In(NO_3)_3$ xH_2O) was purchased from Chem-impex International. All aqueous solutions were prepared using ultrapure water.

Materials synthesis

$Cu(OH)_2$ nanowires were synthesized by continuous precipitation by feeding 0.5 M $Cu(NO_3)_2$ into 1.5 M NaOH (1.0 mL min^{-1}, 3 h) under vigorous stirring at room temperature. The resulting blue precipitate was collected by centrifugation, thoroughly rinsed with deionized water to neutral pH, and dried overnight at 60 °C. Indium was introduced by slowly infusing an aqueous $In(NO_3)_3$ solution into a dispersion of the $Cu(OH)_2$ nanowires (0.10 mL min^{-1}). The superhydrophilic $Cu(OH)_2$ surface enables rapid wetting of the nanowire scaffold and promotes homogeneous adsorption of In species along the nanowires, thereby suppressing precursor agglomeration during the subsequent aging step.[S1,2] After aging 20 h, the solid was collected by centrifugation, washed repeatedly with water to remove residual nitrate, and dried overnight at 60 °C to obtain In_1Cu. Sn_1Cu was prepared analogously by substituting $In(NO_3)_3$ with $SnCl_2$. The Sn/In nanoparticles were prepared by the following method. 6.7 g $SnCl_2$ was dissolved into 100 mL deionized water and magnetically stirred for 120 min at 80 °C. The reaction product was immediately filtered out, washed with deionized water and ethanol, and dried at 60 °C for 12 h in a vacuum oven to obtain Sn nanoparticle. For In nanoparticle synthesis, 3 g $In(NO_3)_3$ was dissolved into 100 mL deionized water and magnetically stirred for 120 min at 80 °C. The reaction product was immediately

filtered out, washed with deionized water and ethanol, and dried at 60 °C for 12 h in a vacuum oven to obtain In nanoparticle.

Electrode Preparation

Electrodes were prepared by airbrushing a catalyst ink onto a gas-diffusion substrate. Briefly, 35 mg of catalyst was suspended in 5 mL of isopropanol and sonicated for 30 min to obtain a homogeneous dispersion. The ink was then spray-coated uniformly onto carbon paper (Sigracet GDL 34BC, Fuel Cell Store; 3.0 × 4.5 cm) placed on a hot plate maintained at 70 °C, affording a catalyst loading of 0.8 mg cm^{-2} after solvent evaporation. The resulting gas-diffusion electrode was subsequently sectioned into six squares (1 × 1 cm) for electrolysis tests.

Electrochemical measurements

CO_2RR measurements as a function of temperature and pressure were conducted in a membrane-electrode assembly (MEA) cell using 0.1 M $KHCO_3$ as the anolyte. The gas-diffusion cathode and an IrO_2/Ti-felt anode were separated by a PiperION anion-exchange membrane (AEM, 20 µm, Fuel Cell Store). For the long-term stability measurements, we employed a 40 µm AEM, which enabled longer operation. Dry CO_2 was delivered to the cathode at 50 sccm using a mass-flow controller (Alicat Scientific). Electrolysis was performed in galvanostatic mode using a potentiostat (Gamry Interface 5000E), while the full-cell voltage was recorded without iR compensation. Cell temperature was regulated by electrical heating rods integrated with both cathode and anode flow fields, with a thermocouple embedded in the cell providing feedback to a PID controller (Cole-Parmer TC5000) to maintain the setpoint. Cathode and anode pressures were matched throughout operation. The anode pressure was set by a downstream back-pressure regulator (Equilibar LF2, PEEK non-reinforced diaphragm) driven by a high-pressure electronic pilot controller (Equilibar). Anolyte was supplied to the anode compartment using a high-pressure syringe pump (Fusion 6000X, Chemyx) at 0.5 mL min^{-1}. The cathode pressure was controlled with a series of back-pressure

regulators (Swagelok, KBP1J0A4A5A20000). A cold trap placed downstream of the cathode outlet enabled phase separation of gas and condensable products. Liquid-phase faradaic efficiencies were determined from the combined collection of anode- and cathode-side liquids over identical time intervals. Gas products were analyzed downstream of the pressure regulation stage, after depressurization to ambient pressure. HER measurements were conducted in a two-compartment H-cell separated by a proton-exchange-membrane. The catalyst inks were drop-cast onto glassy carbon working electrodes and dried under ambient conditions prior to electrochemical testing. An Ag/AgCl electrode was used as the reference electrode, while a platinum foil served as the counter electrode. All HER measurements were performed in Ar-saturated electrolyte. Linear sweep voltammetry was carried out by sweeping the potential from 0 to -1.5 V vs. RHE at a scan rate of 20 mV s^{-1}.

Product Analysis

Gaseous products were quantified online using an in-line gas chromatograph (Agilent 8860). To determine the cathode outlet flow rate required for FE calculations, the CO_2 effluent stream was measured upstream of the GC sampling loop using a mass flow meter (Alicat Scientific).[S3] Gas-phase faradaic efficiencies were calculated as:

$$FE(\%) = \frac{zFxV}{j_{total}} \times 100\%$$

where z is the number of electrons transferred for producing a target product; F is the Faraday constant; x is the molar fraction of a target product determined by GC; V is the molar flow rate of gas; and j_{total} is the total current density. Liquid products were collected after electrolysis and analyzed by ^{1}H NMR (Bruker NEO, 400 MHz). For quantification, 500 μL of electrolyte was combined with 100 μL of an internal-standard solution (5 mM 3-(trimethylsilyl)propionic-2,2,3,3-d_4 acid sodium salt in D_2O). Partial current densities were obtained from $j_i = j_{\text{total}} \times \text{FE}_i$ at each operating condition.

In situ ATR-SEIRAS

Prior to ATR-SEIRAS measurements, the Si prism substrate was cleaned and

functionalized to yield a reproducible Au-enhanced interface. The prism was first immersed in aqua regia to remove surface contaminants, then mechanically polished with a 0.05 μm Al_2O_3 slurry. Residual particles were removed by sequential ultrasonication in DI water, acetone, and DI water (5 min each). After drying, a thin Au layer was deposited by sputtering (Hitachi E-1010) for 60 s using an Au target. In situ ATR-SEIRAS measurements were conducted in a three-electrode, sandwich-type spectroelectrochemical cell, with the Au-coated Si prism as the working electrode, a Pt bar as the counter electrode, and an Ag/AgCl as the reference electrode. The cell was coupled to a Thermo Fisher Nicolet iS50 FTIR spectrometer equipped with a liquid-nitrogen-cooled MCT detector. The electrolyte temperature was regulated using an external circulating water bath. CO_2 was continuously bubbled through 0.1 M $KHCO_3$ to maintain CO_2 saturation during measurements. Spectra were collected with 16 co-added scans at a resolution of 4 cm^{-1} and referenced to a background recorded at 0.1 V versus RHE in CO_2-saturated 0.1 M $KHCO_3$. All potential calibration experiments were carried out in a PINE standard five-neck electrochemical cell equipped with a water jacket for precise temperature control, using a PINE rotating ring-disk electrode (RRDE) setup. For each target temperature, the electrolyte was first equilibrated in the water-jacketed cell until the temperature was stabilized within ±1 °C. The solution was then purged with high-purity H_2 for at least 20 min to establish H_2 saturation. Under these conditions, the open-circuit potential between the Pt working electrode and the Ag/AgCl reference electrode was measured at each temperature individually, thereby determining the potential offset required to convert the measured potentials to the RHE scale for that specific temperature and electrolyte. For the ATR-SEIRAS measurements, iR compensation was performed after the cell had reached the preset temperature and gas saturation state and before spectral acquisition. Specifically, automatic iR compensation (80%) was applied using the CHI 760e electrochemical workstation under each measurement condition. During the in situ infrared experiments, the potential was stepped and held for 10 s for signal collection at each point. Therefore, the reported potentials for the temperature-dependent ATR-SEIRAS comparison were

referenced to temperature-calibrated RHE values and measured under individually compensated solution resistance at each temperature. ATR-SEIRAS spectra were processed using Thermo Scientific OMNIC software. The automatic baseline correction function in the data-processing module was applied over the displayed spectral window to subtract slowly varying background signals and minimize baseline curvature.

Analysis of the O-H stretching region

Specifically, the O-H stretching region (2800-3800 cm^{-1}) was analyzed in OriginPro using the Peak Analyzer (Fit Peaks Pro) module. A user-defined linear baseline was constructed by selecting anchor points from the relatively flat spectral regions near 2800 and 3800 cm^{-1}, followed by baseline subtraction. The resulting envelope was then fitted with Gaussian components centered at 3230, 3417, and 3552 cm^{-1}. To ensure consistency across datasets, the peak centers were fixed, whereas the peak areas and widths were optimized by nonlinear least-squares fitting until convergence.

Material characterization

X-ray diffraction (XRD) patterns were performed on a Panalytical Empyrean diffractometer at an operation voltage of 45 kV and a current of 40 mA, and catalysts in powder form were directly measured. The X-ray photoelectron spectroscopy (XPS) spectra of powder materials and post-sample on carbon papers were executed and obtained by a Thermo Scientific (Waltham, MA, USA) Model K-Alpha XPS instrument utilizing monochromatic Al Kα X-rays of 1486.6 eV. Scanning electron microscopy (SEM) and energy dispersive X-ray spectroscopy (EDS) characterizations of the powder materials and catalyst coated carbon papers were carried out with a ThermoFisher Scientific Scios 2 Dual Beam Focused Ion Beam at 5 and 20 kV, respectively. Aberration-corrected scanning transmission electron microscopy (STEM) using a JEOL NEOARM and equipped with dual 100 mm^2 solid state detectors for energy dispersive X-ray spectroscopy (EDS) was employed to characterize the catalyst morphology and compositions. STEM images were obtained at 200 kV, and the

corresponding STEM-EDS maps were achieved and processed in the JEOL Analysis Station software using the standard-less Cliff-Lorimer method. Inductively coupled plasma mass spectrometry (ICP-MS) determination was performed with the quadrupole model iCAP-RQ (ThermoFisher Scientific, Bremen) in a standard mode, utilizing QTegra® ISDS software and linear regression. The powder materials and catalyst coated carbon papers were dissolved in 4 mL of aqua regia. Subsequently, 0.5 mL of the dissolved aqua regia solution was diluted into 5 mL- with DI water for ICP-MS detection. X-ray absorption Spectroscopy (XAFS) was performed on ex situ samples at the Quick X-ray absorption and scattering (7-BM) beamline. Samples on carbon paper were sealed with Kapton tape and stacked to increase signal quality, with spectra being collected in fluorescence mode using a PIPS detector. Reference foil spectra were collected in tandem via a reference ionization detector for each scan using Cu, In, and Sn foil for the Cu K Edge, In Edge, and Sn K Edge, respectively, and used for energy calibrations. Data analysis was carried out via the Demeter Athena/Artemis package for XANES/EXAFS, respectively, with Cu, In, and Sn foils used to determine S_0^2 for either respective edge.

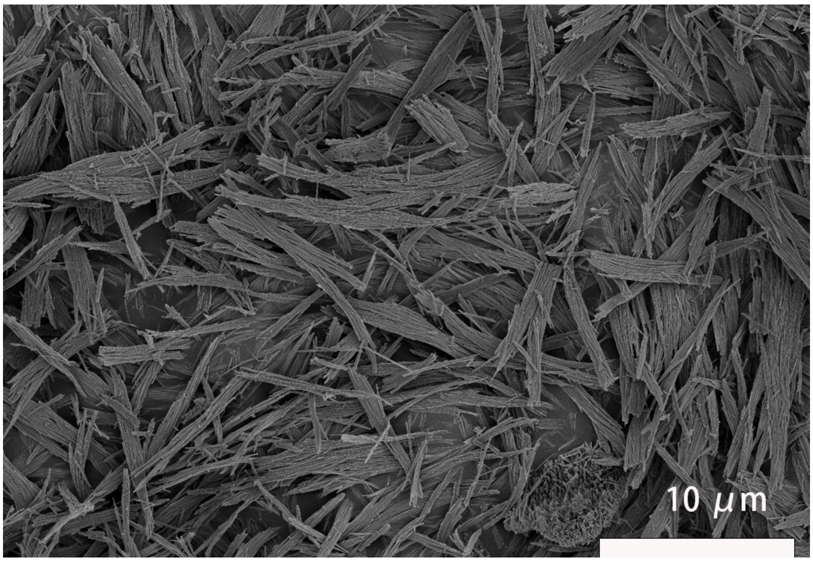

Figure S1. SEM image of $Cu(OH)_2$ powders.

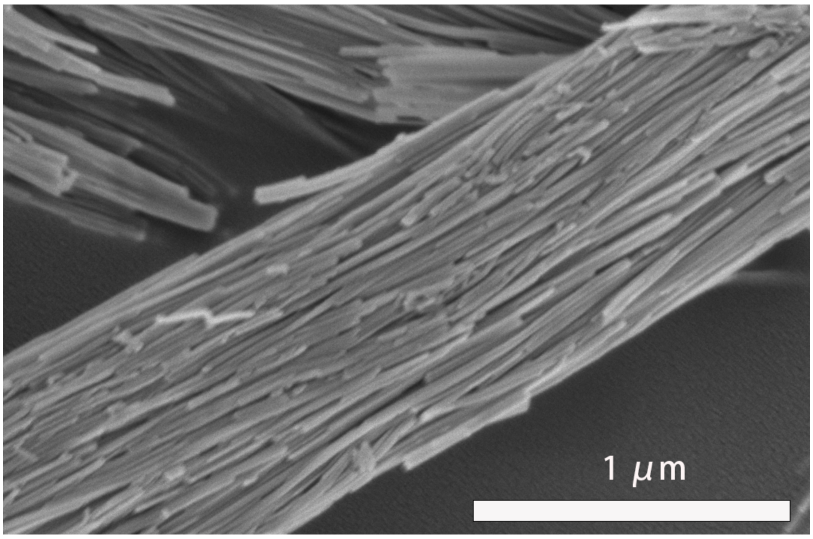

Figure S2. SEM image of the pre-In_1Cu catalysts.

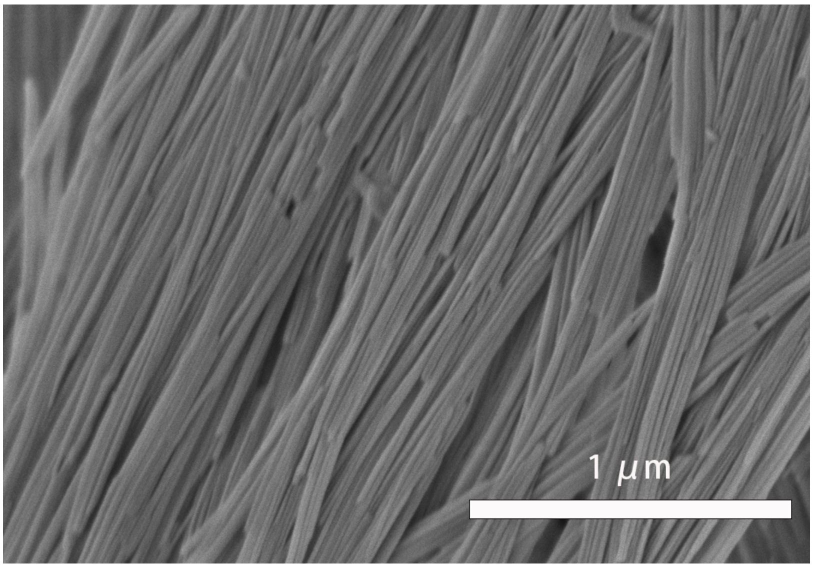

Figure S3. SEM image of pre-Sn_1Cu catalysts.

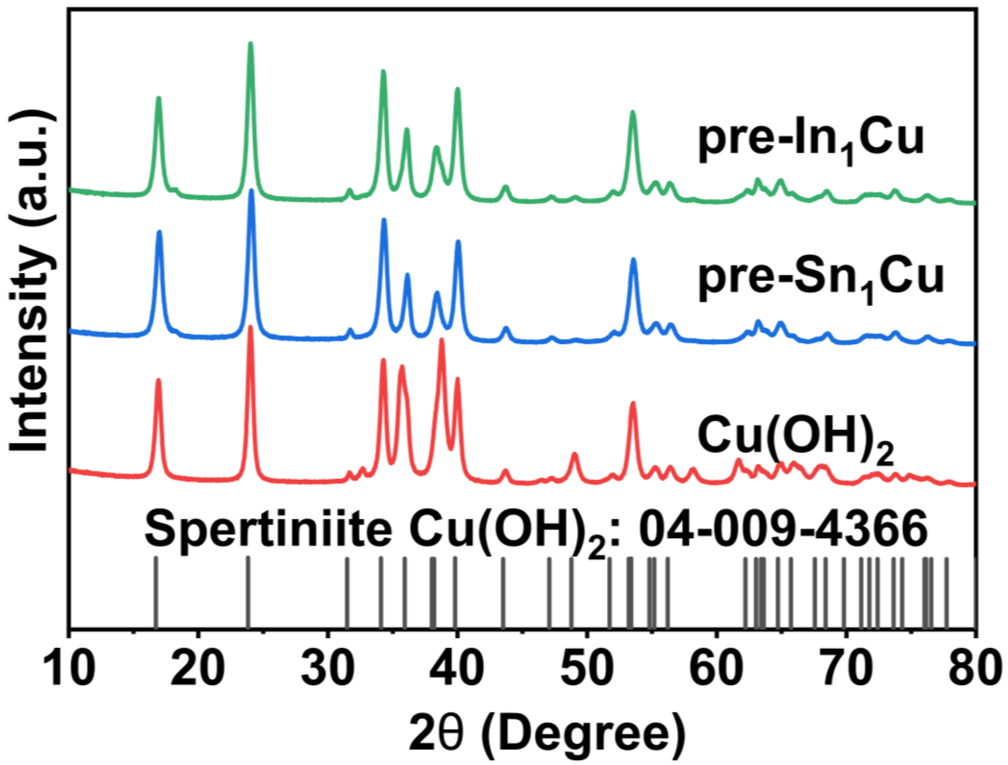

Figure S4. XRD patterns of the $Cu(OH)_2$, pre-In_1Cu and pre-Sn_1Cu catalysts.

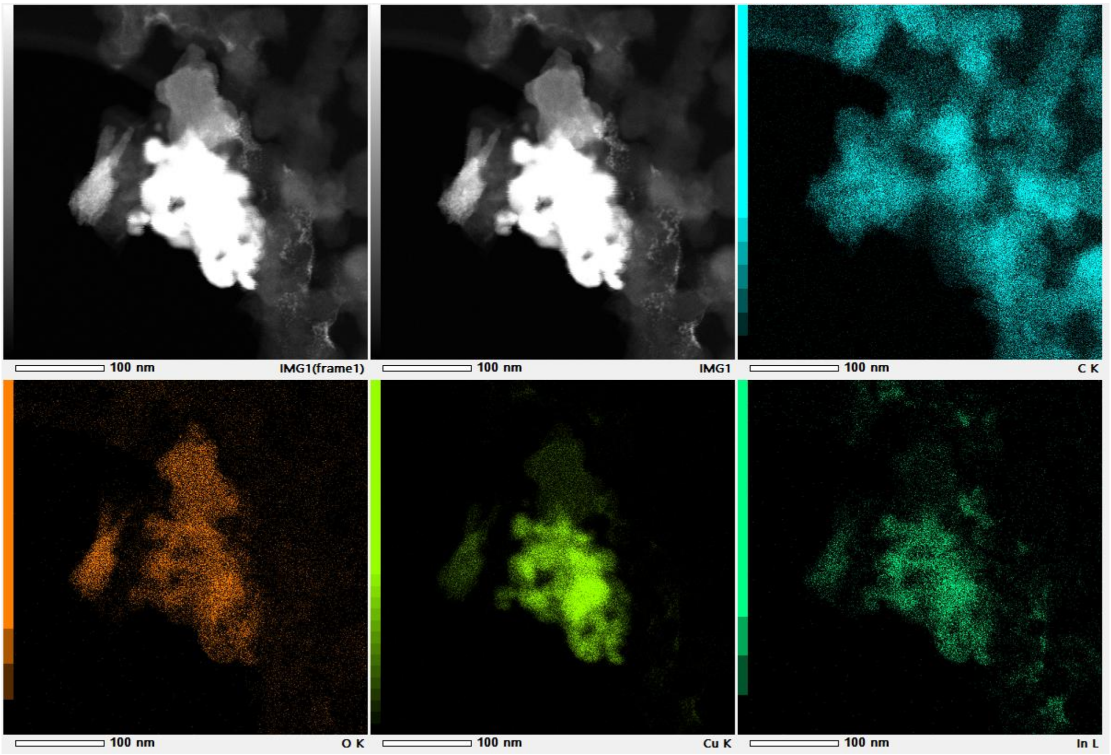

Figure S5. Elemental distribution of pre-In_1Cu revealed by STEM-EDS.

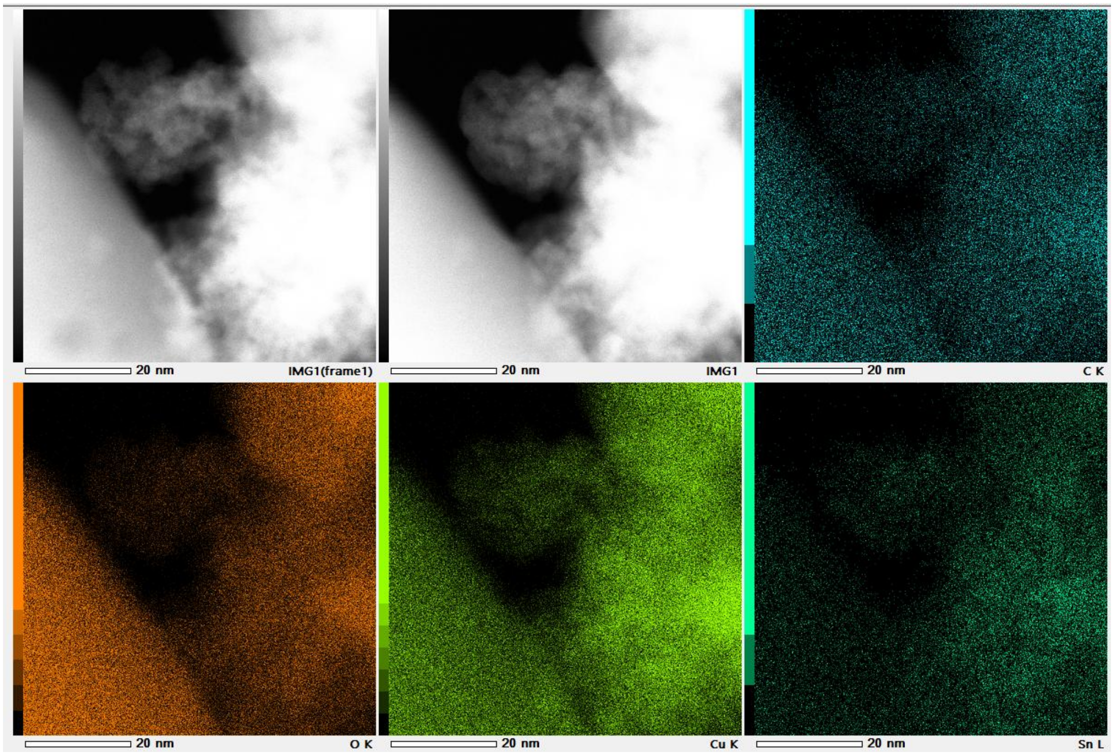

Figure S6. Elemental distribution of pre-Sn_1Cu revealed by STEM-EDS.

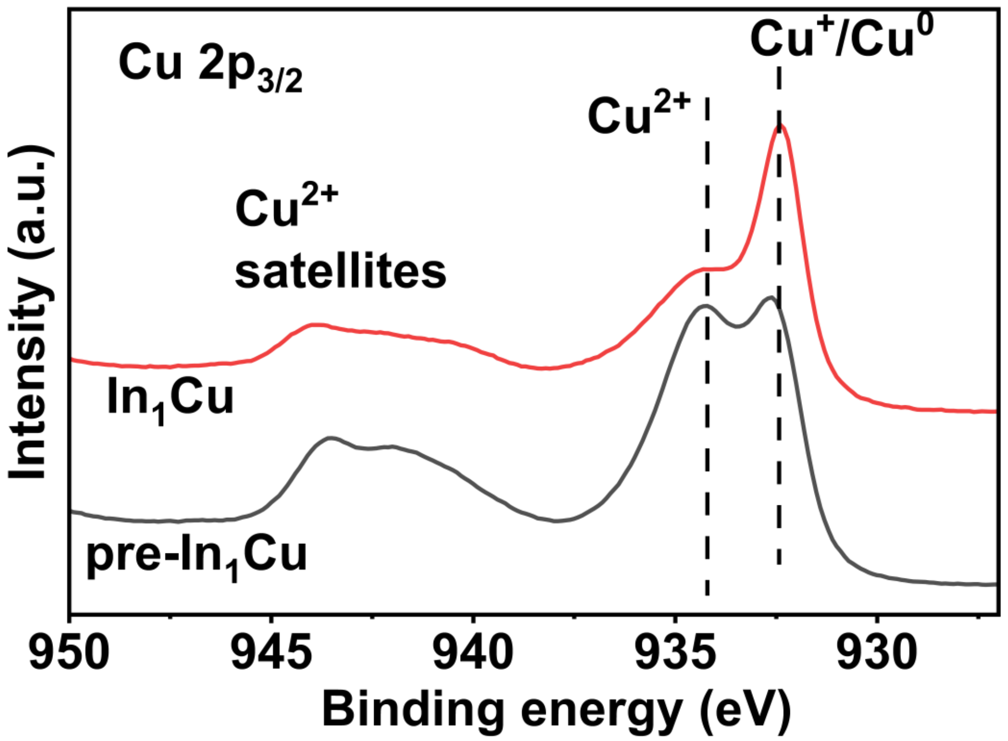

Figure S7. High-resolution Cu 2p XPS spectra of pre-In_1Cu and In_1Cu.

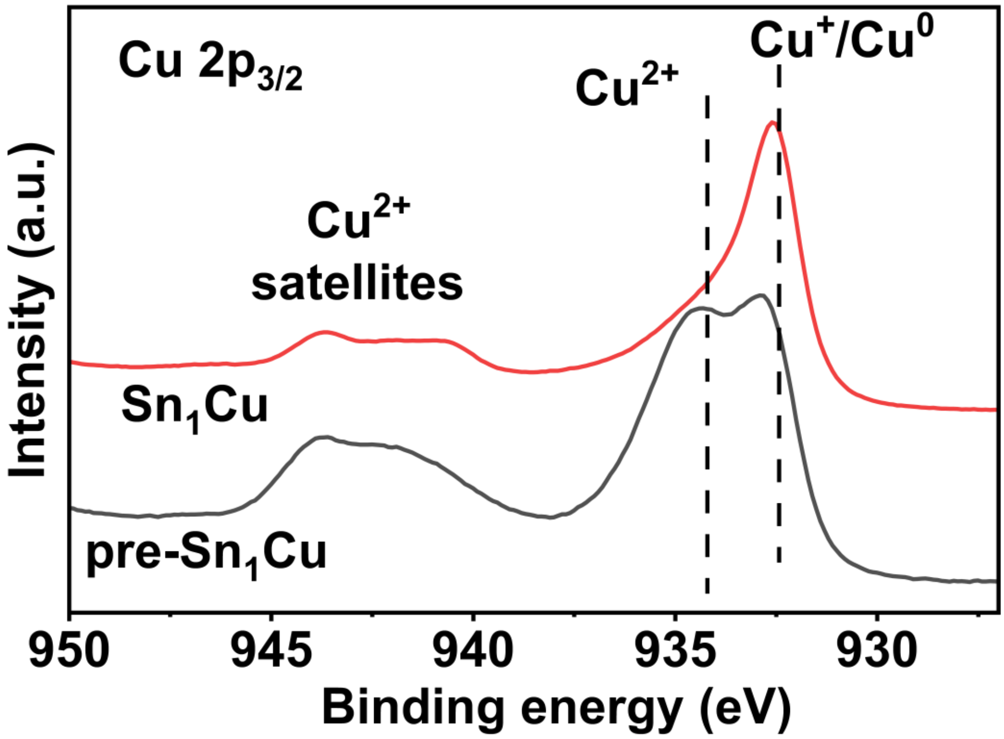

Figure S8. High-resolution Cu 2p XPS spectra of pre-Sn_1Cu and Sn_1Cu.

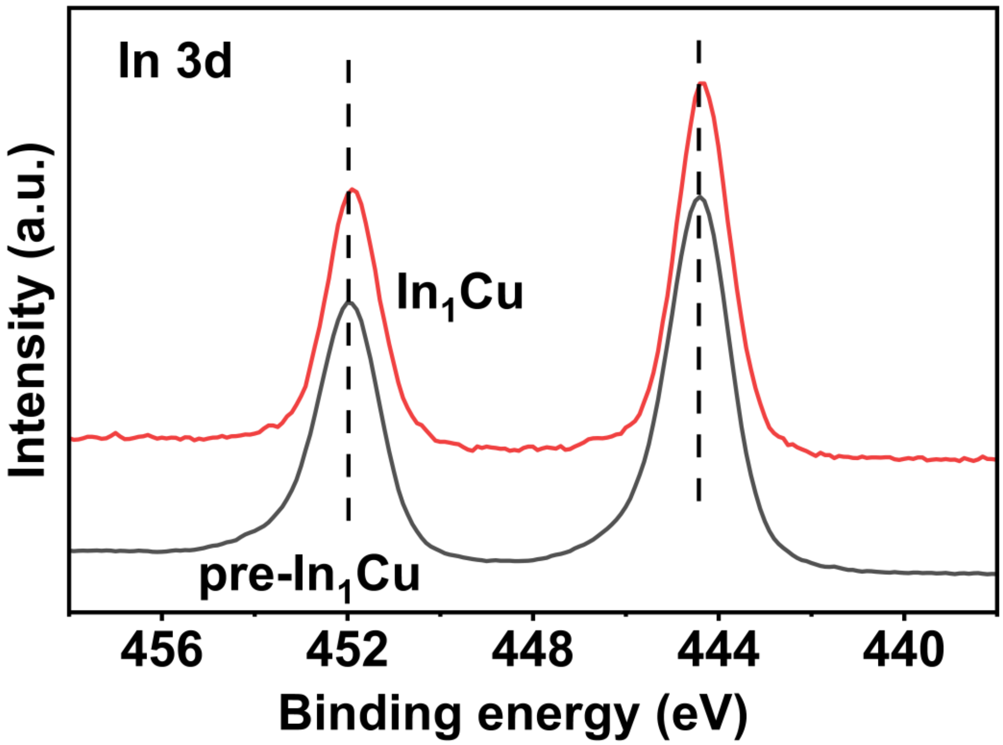

Figure S9. High-resolution In 3d XPS spectra of pre-In_1Cu and In_1Cu.

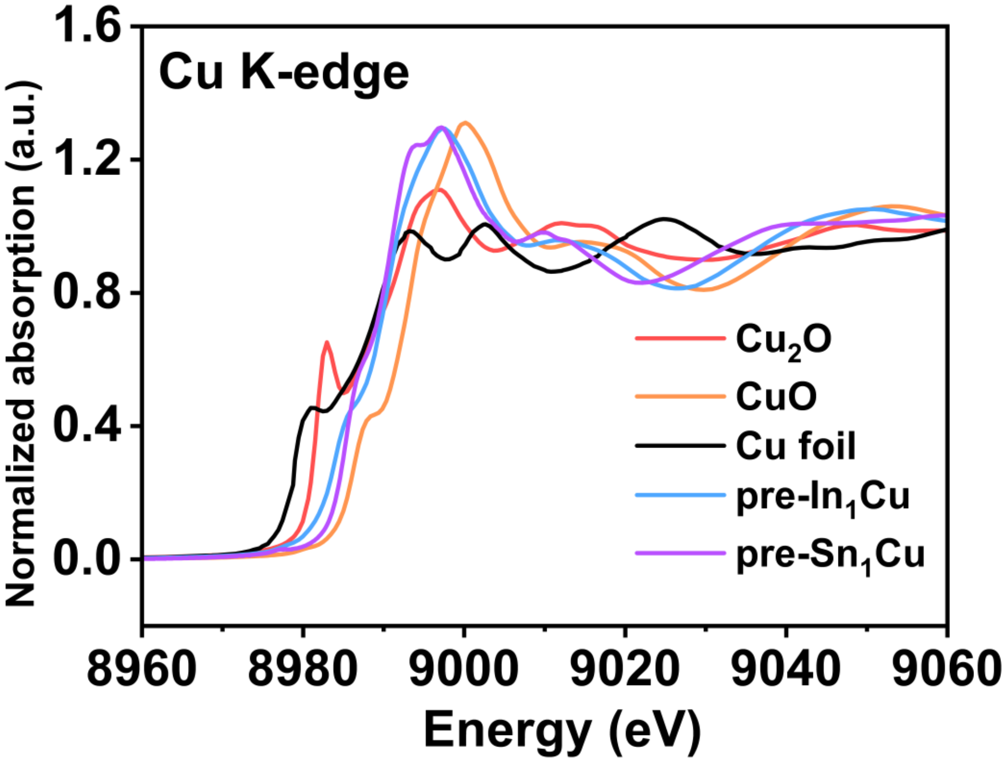

Figure S10. Cu K-edge XANES spectra of pre-In_1Cu and pre-Sn_1Cu, benchmarked by Cu foil, Cu_2O, and CuO.

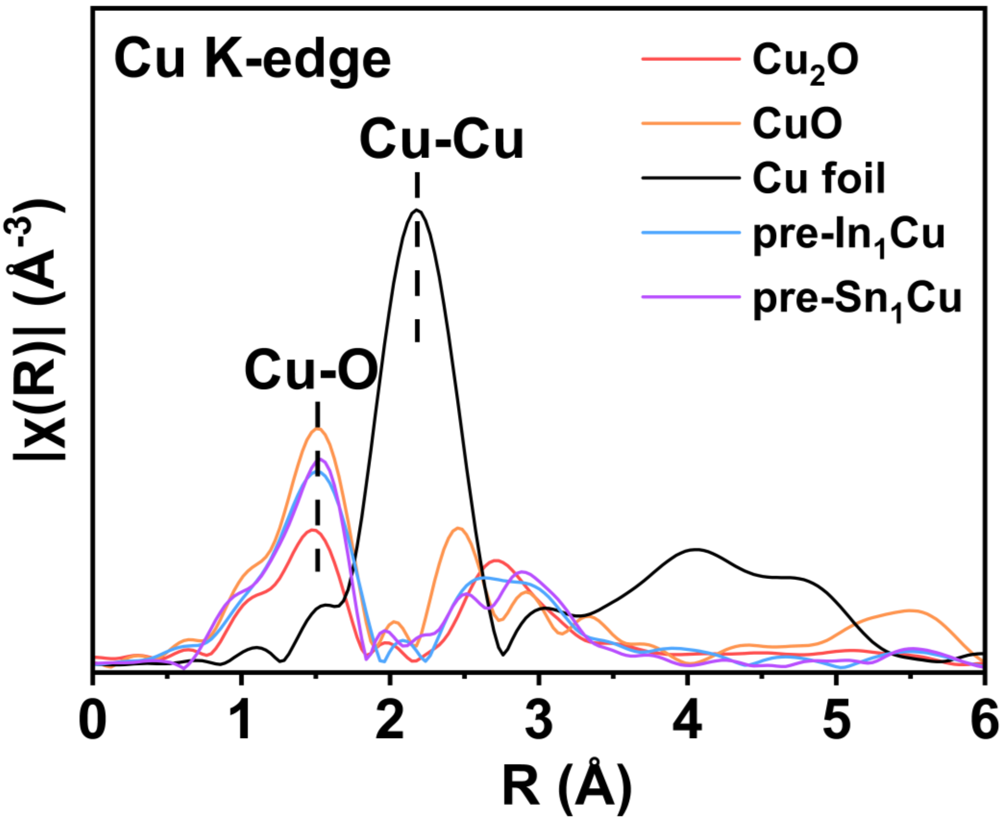

Figure S11. FT-EXAFS spectra at Cu K-edge of pre-In_1Cu and pre-Sn_1Cu, benchmarked by Cu foil, Cu_2O, and CuO.

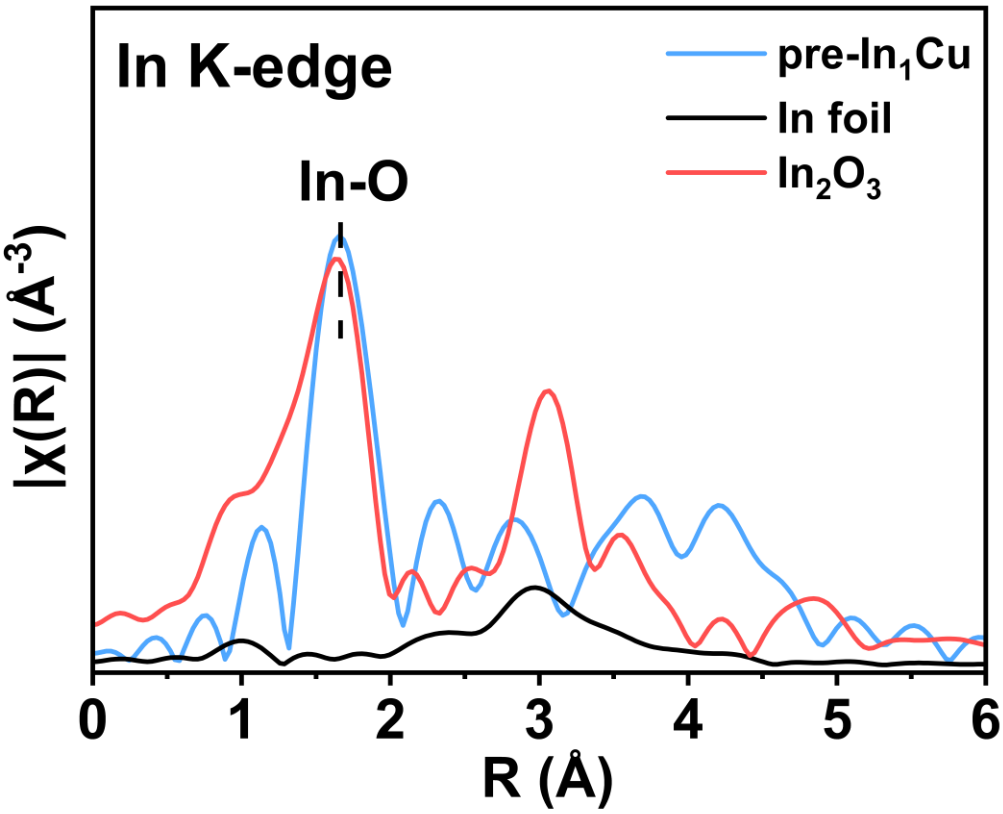

Figure S12. In K-edge FT-EXAFS spectra of pre-In_1Cu, benchmarked by In foil and In_2O_3.

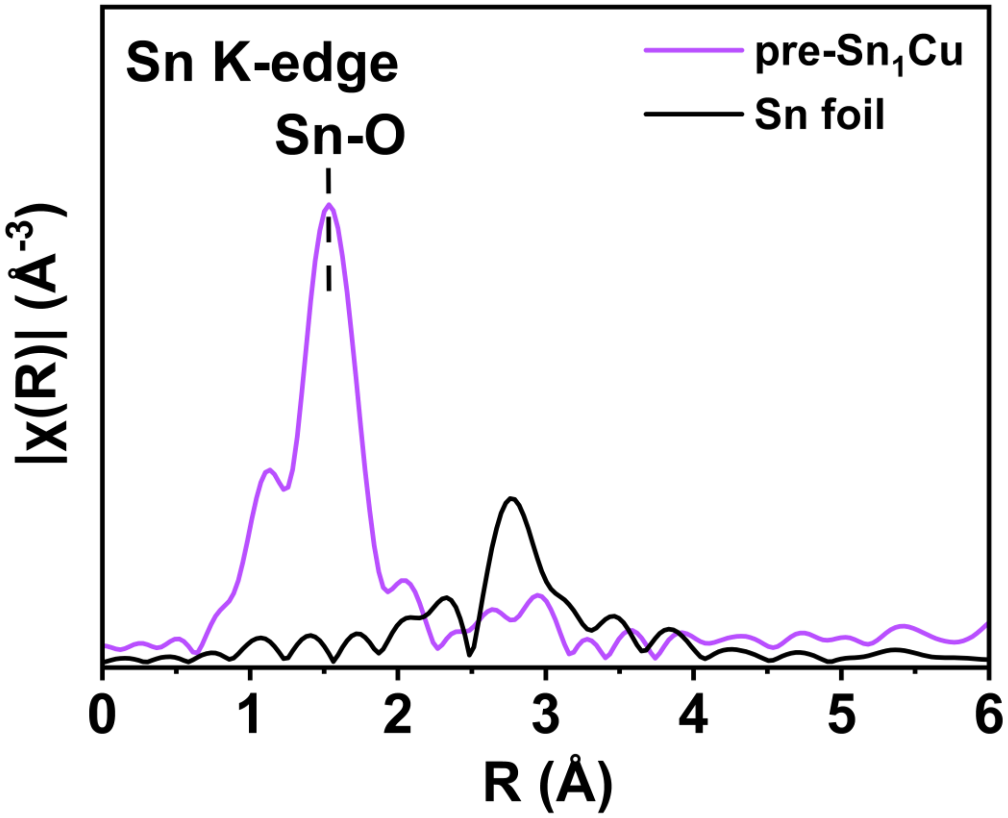

Figure S13. Sn K-edge FT-EXAFS spectra of pre-Sn_1Cu, benchmarked by Sn foil.

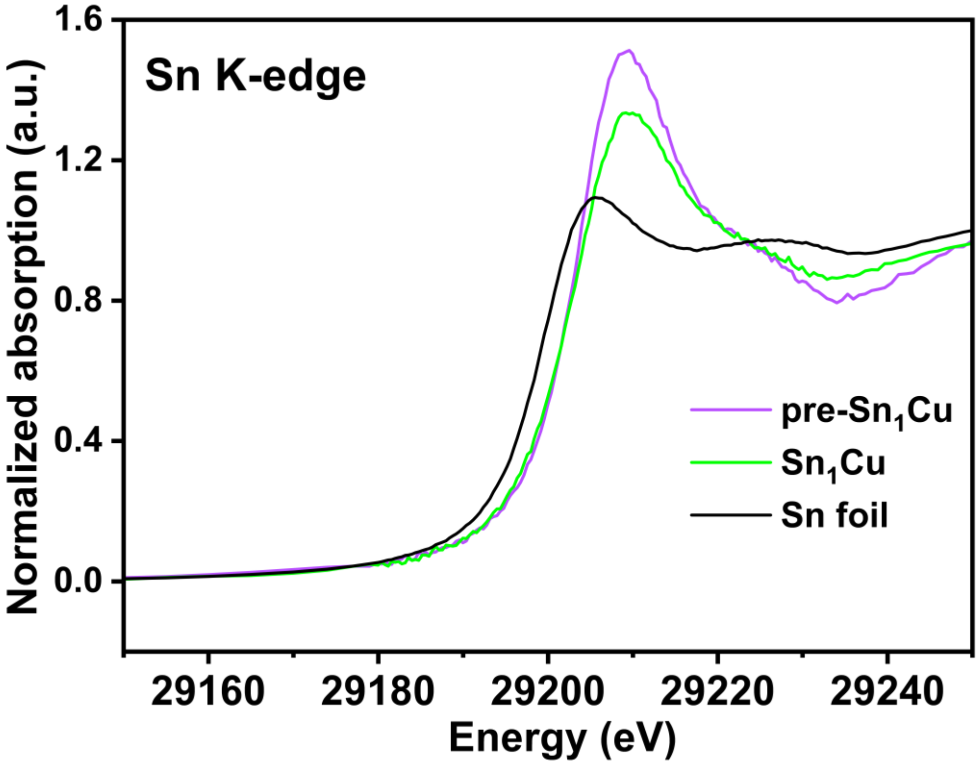

Figure S14. Sn K-edge XANES spectra of pre-Sn_1Cu and Sn_1Cu, benchmarked by Sn foil.

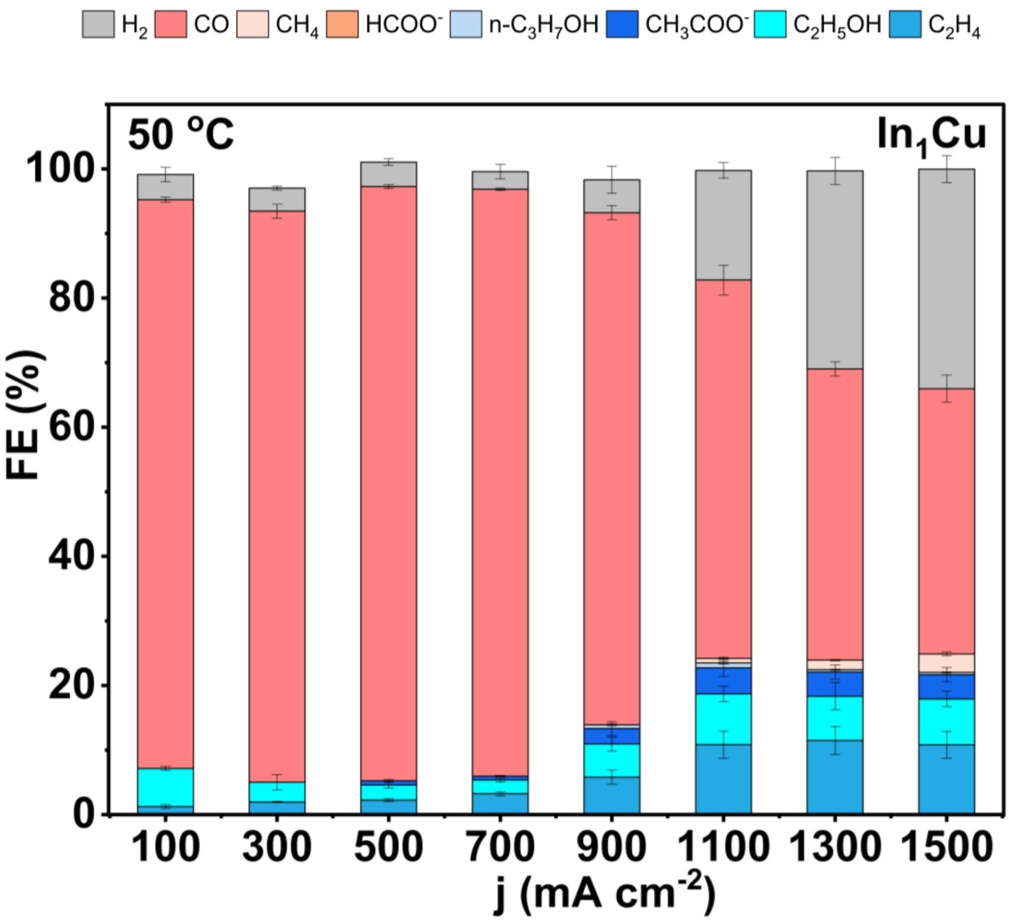

Figure S15. FE for all products on In_1Cu at 50 °C as a function of current density (100-1,500 mA cm^{-2}). Error bars represent the standard deviation from three independent measurements (n =3).

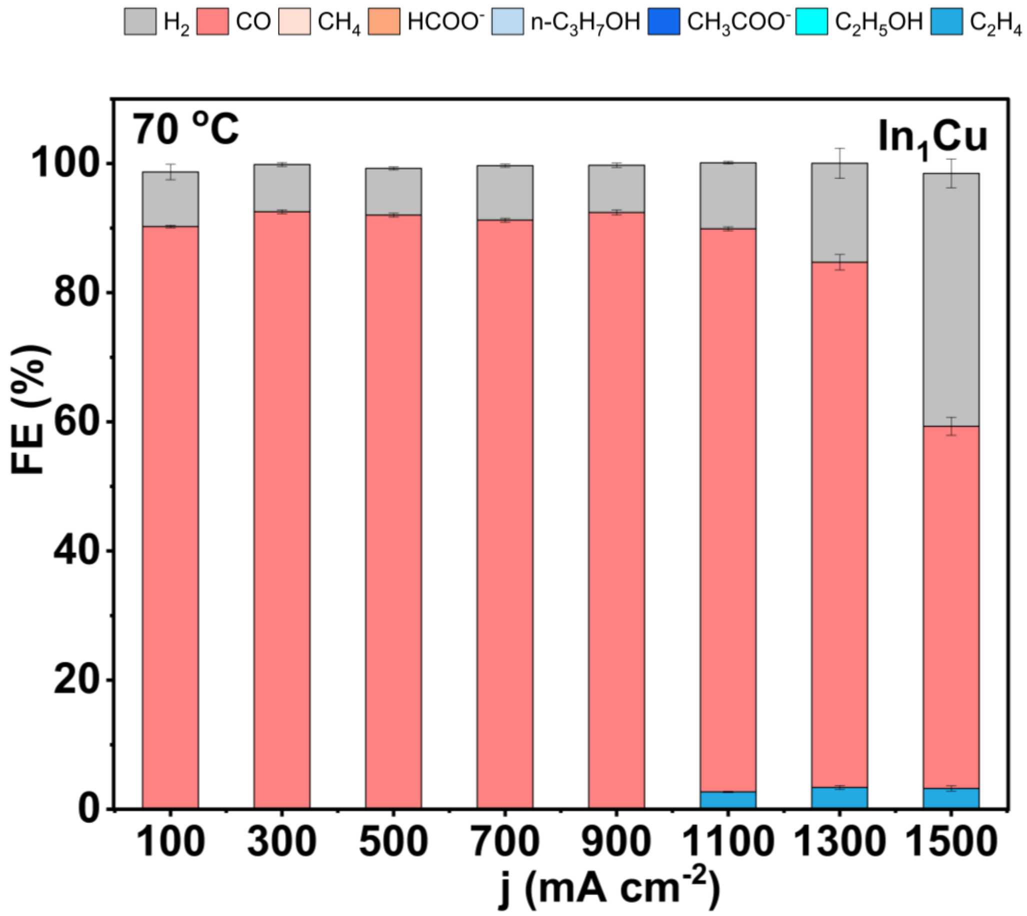

Figure S16. FE for all products on In_1Cu at 70 °C as a function of current density (100-1,500 mA cm^{-2}). Error bars represent the standard deviation from three independent measurements (n =3).

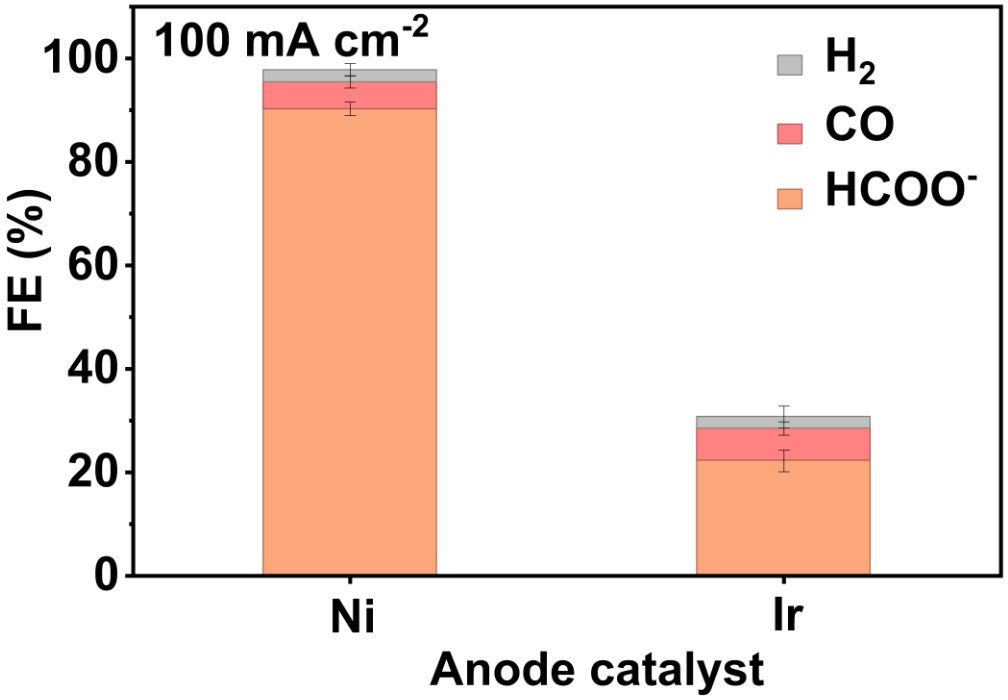

Figure S17. Product distribution of Sn as cathode catalyst at 100 mA cm^{-2}, with Ni or Ir as the anode catalyst, respectively. Error bars represent the standard deviation from three independent measurements (n =3).

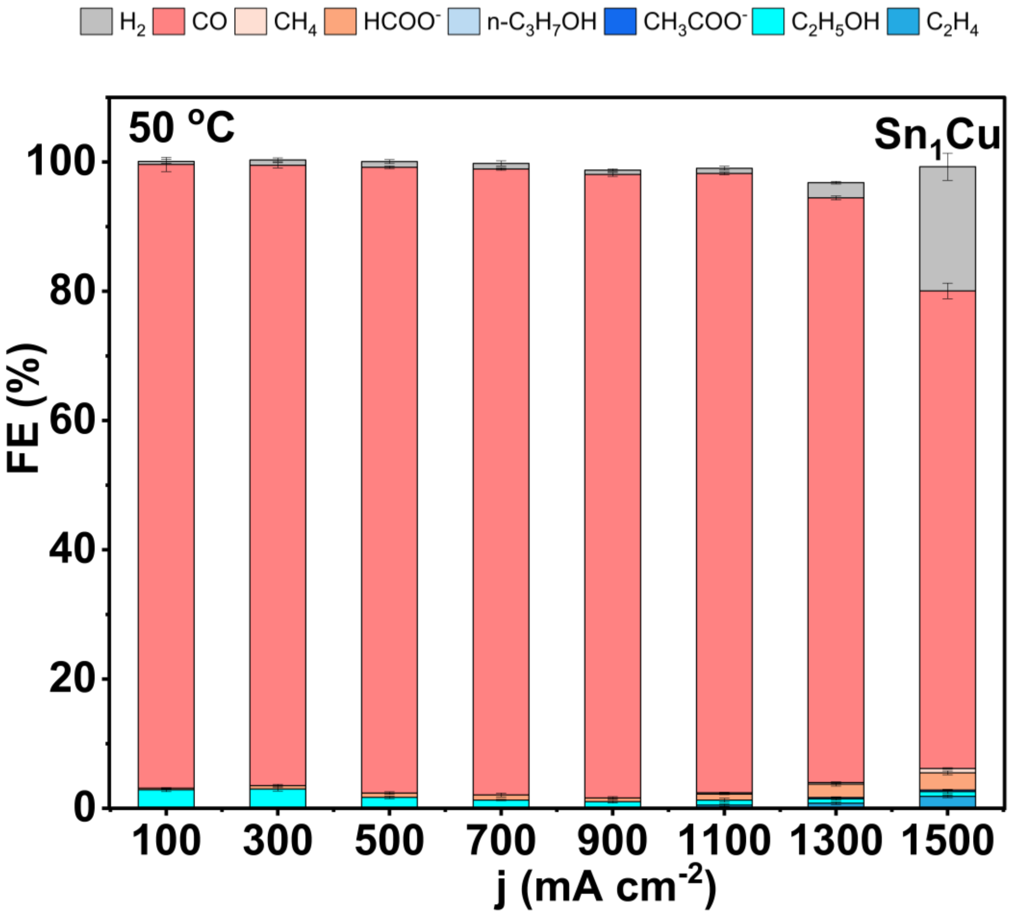

Figure S18. FE for all products on Sn_1Cu at 50 °C as a function of current density (100-1,500 mA cm^{-2}). Error bars represent the standard deviation from three independent measurements (n =3).

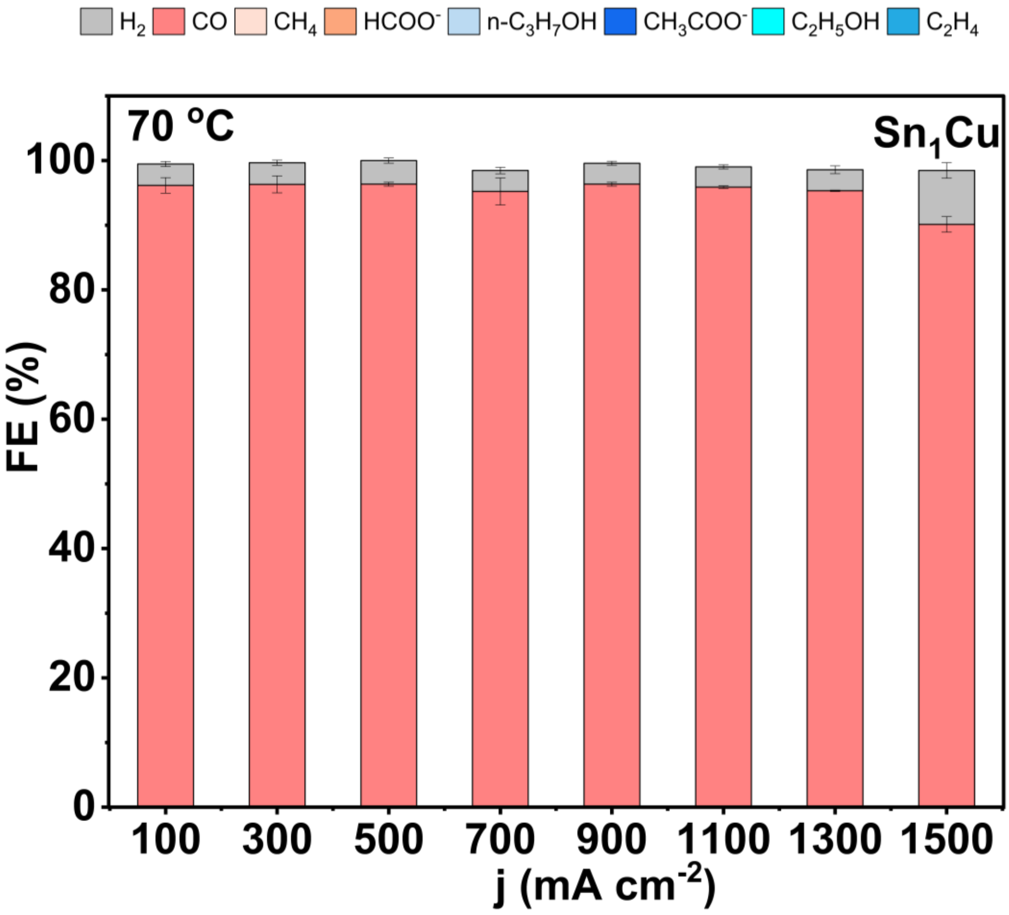

Figure S19. FE for all products on Sn_1Cu at 70 °C as a function of current density (100-1,500 mA cm^{-2}). Error bars represent the standard deviation from three independent measurements (n =3).

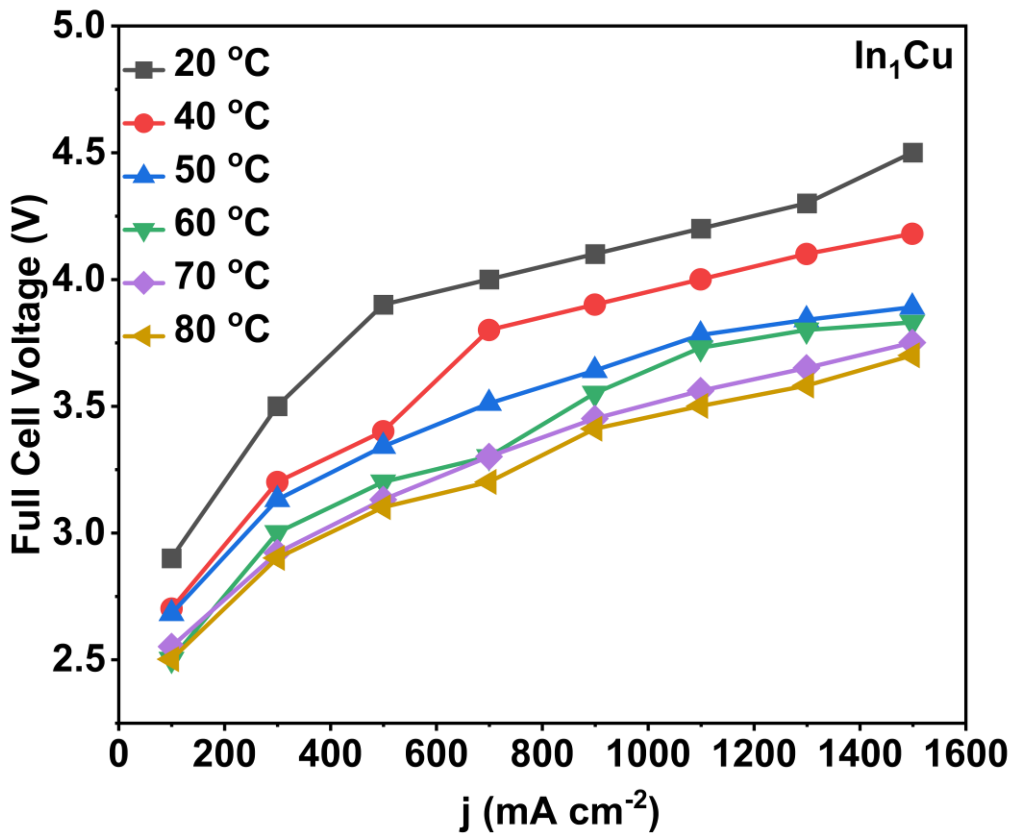

Figure S20. Full cell voltage for In_1Cu at different temperatures from 20 to 80 °C.

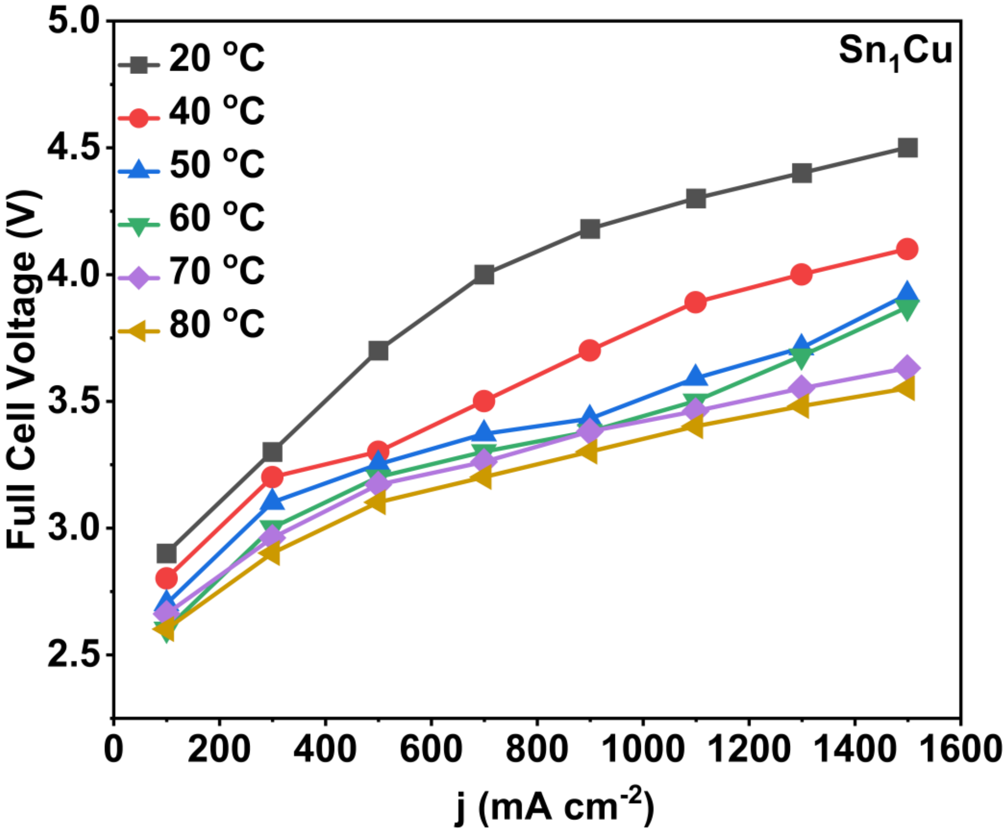

Figure S21. Full cell voltage for Sn_1Cu at different temperatures from 20 to 80 ^oC.

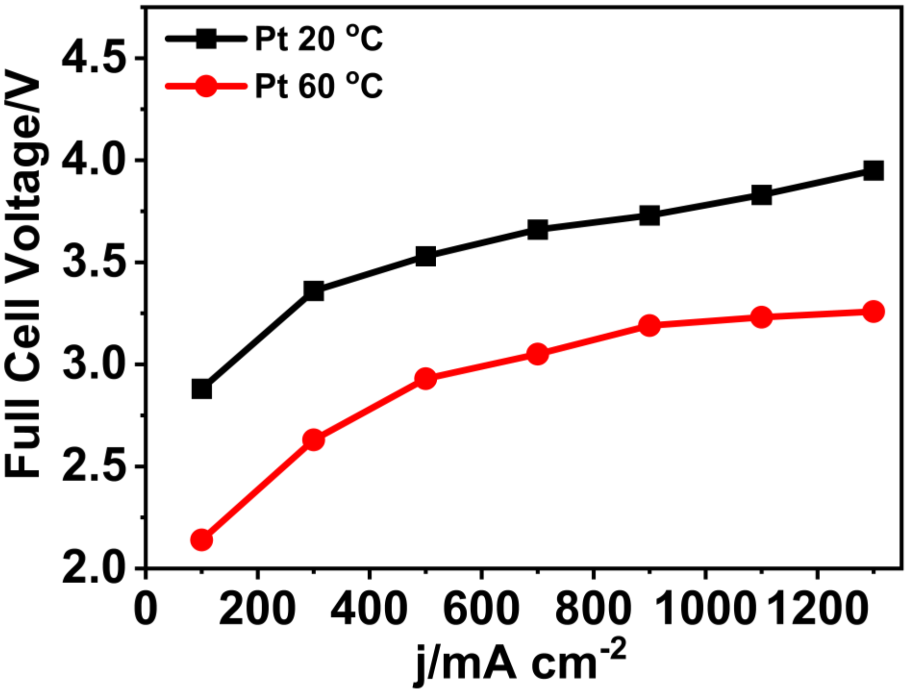

Figure S22. Full cell voltage for Pt cathode at 20 and 60 °C in an MEA cell using 0.1 M $KHCO_3$ as the anolyte.

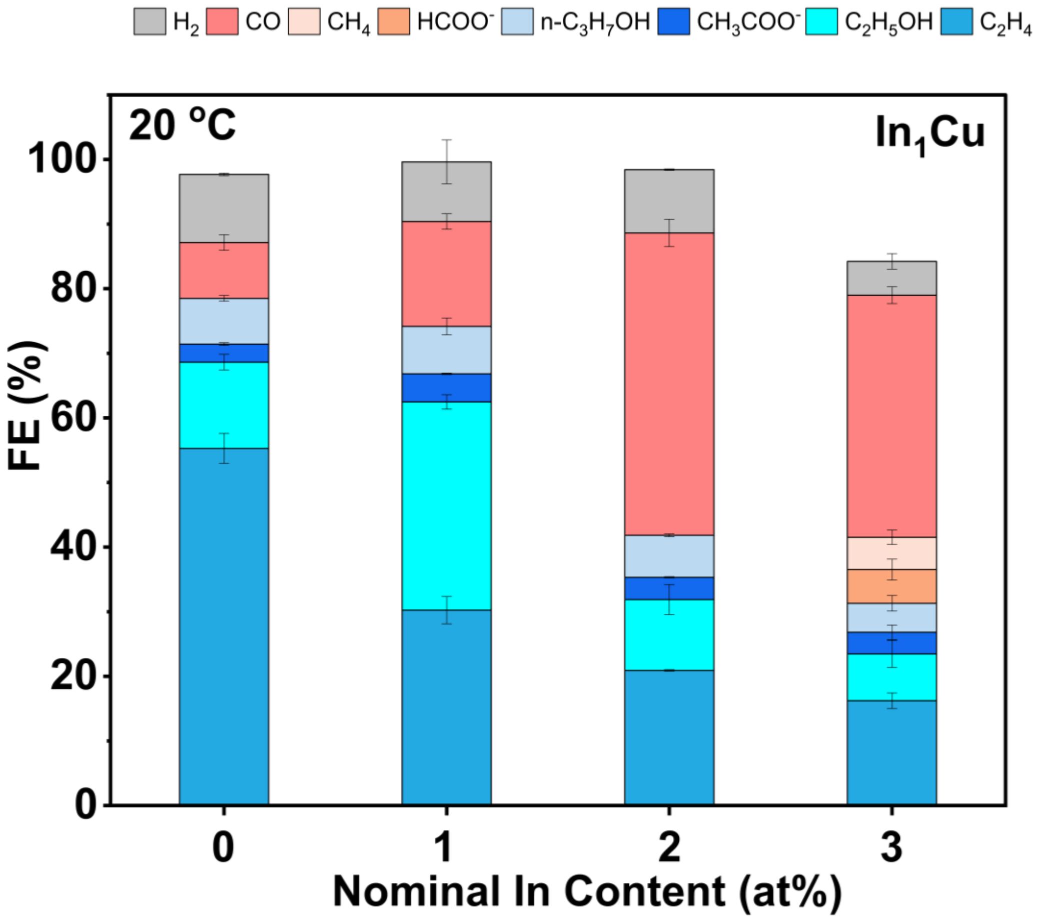

Figure S23. FE for all products on In_1Cu with different nominal In contents at 300 mA cm^{-2} and 20 °C. Error bars represent the standard deviation from three independent measurements (n =3).

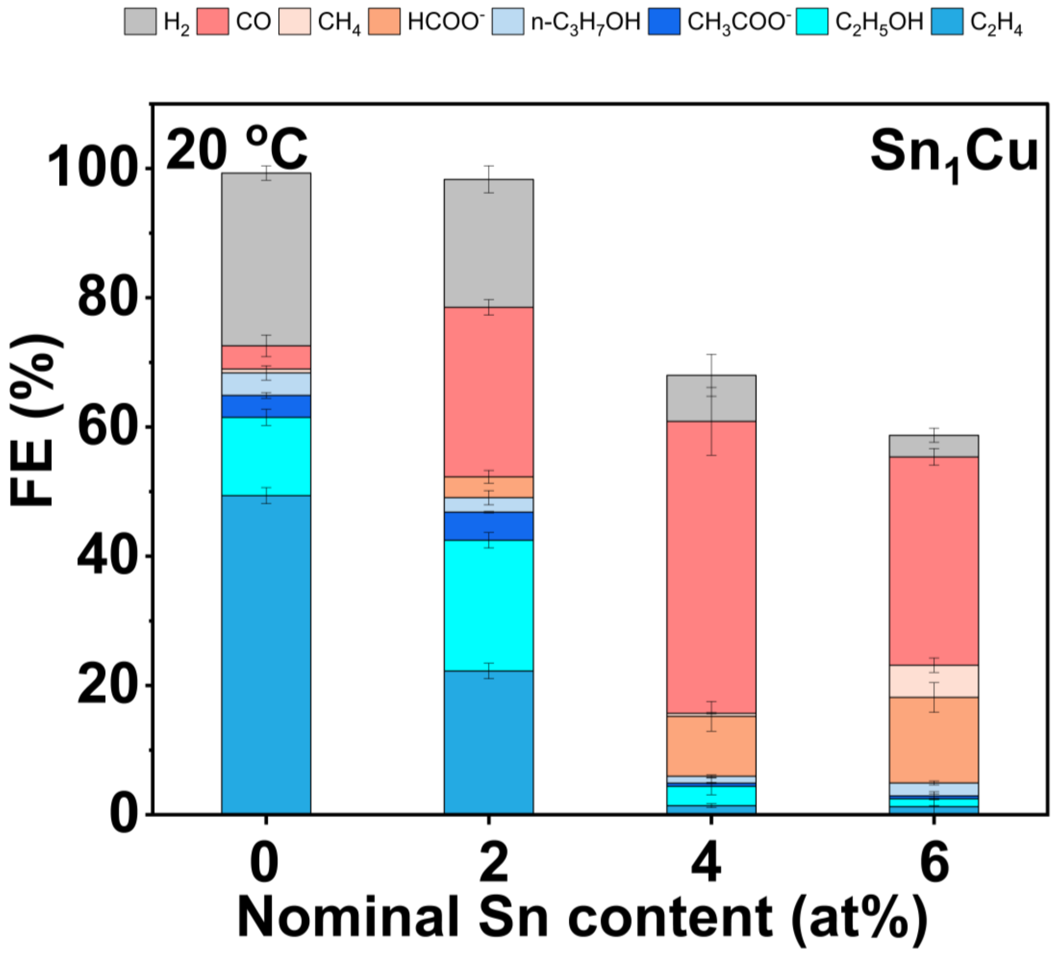

Figure S24. FE for all products on Sn_1Cu with different nominal Sn contents at 500 mA cm^{-2} and 20 °C. Error bars represent the standard deviation from three independent measurements (n =3).

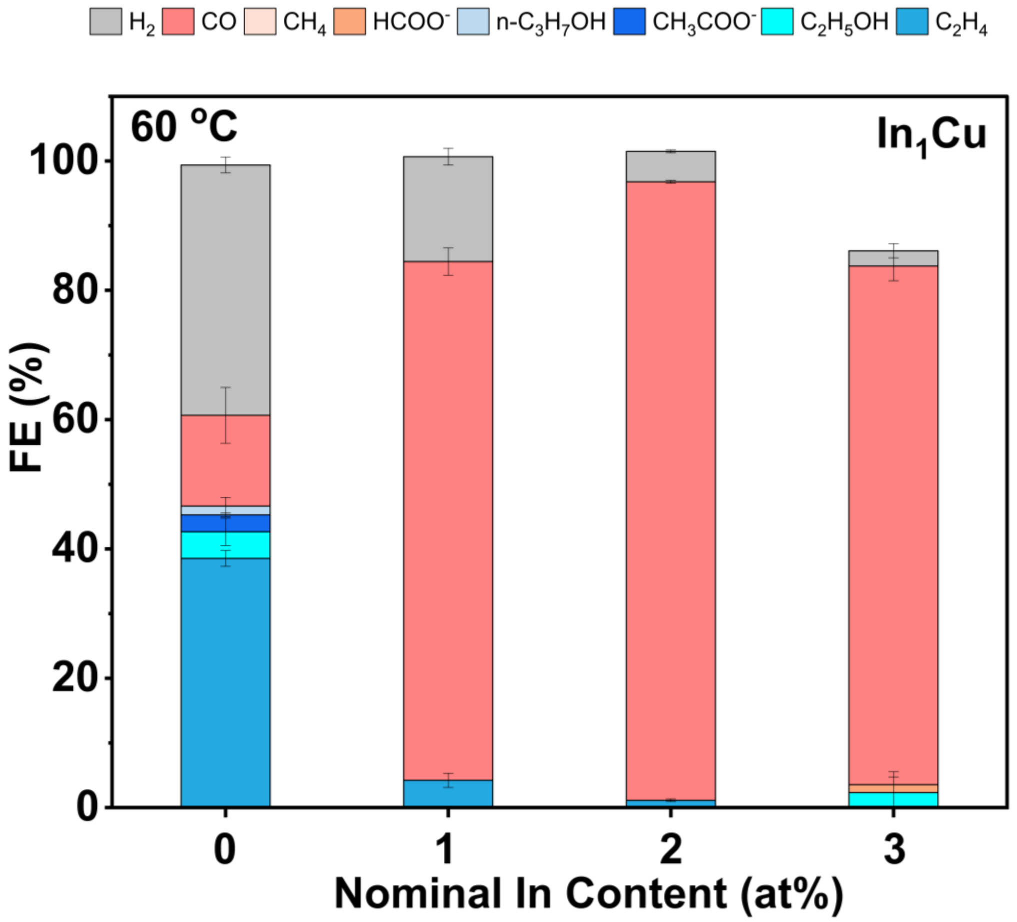

Figure S25. FE for all products on In_1Cu with different nominal In contents at 1,100 mA cm^{-2} and 60 ^oC. Error bars represent the standard deviation from three independent measurements (n =3).

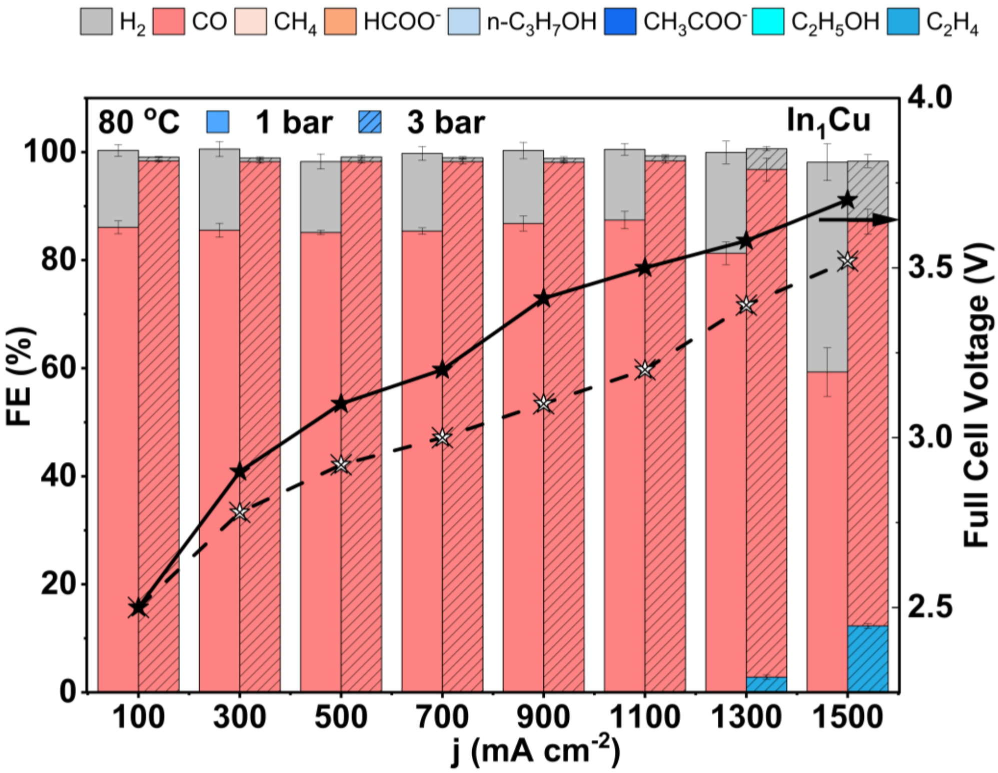

Figure S26. Product distribution on In_1Cu under different current densities at 80 °C with 1 bar and 3 bar. Error bars represent the standard deviation from three independent measurements (n =3).

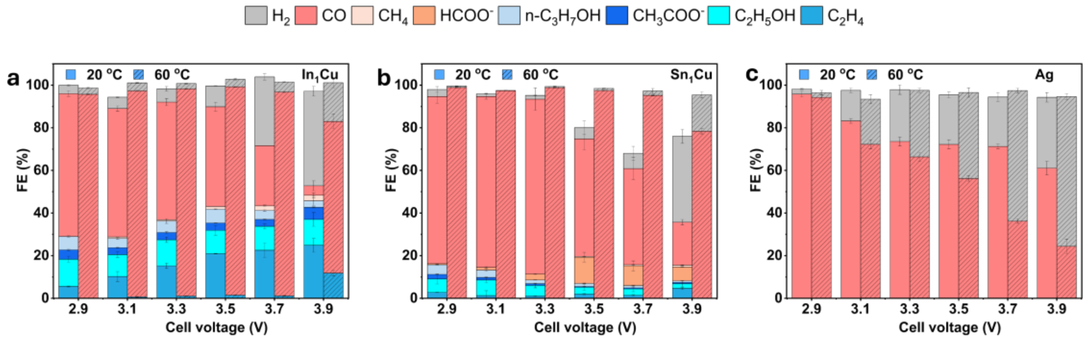

Figure S27. (a) Product distribution of CO_2 reduction on In_1Cu under 20 °C and 60 °C from cell voltage of 2.9 V to 3.9 V. (b) Product distribution of CO_2 reduction on Sn_1Cu under 20 °C and 60 °C from cell voltage of 2.9 V to 3.9 V. (c) Product distribution of CO_2 reduction on Ag under 20 °C and 60 °C from cell voltage of 2.9 V to 3.9 V. The FEs are mean values, while error bars represent the standard deviation from three independent measurements (n =3).

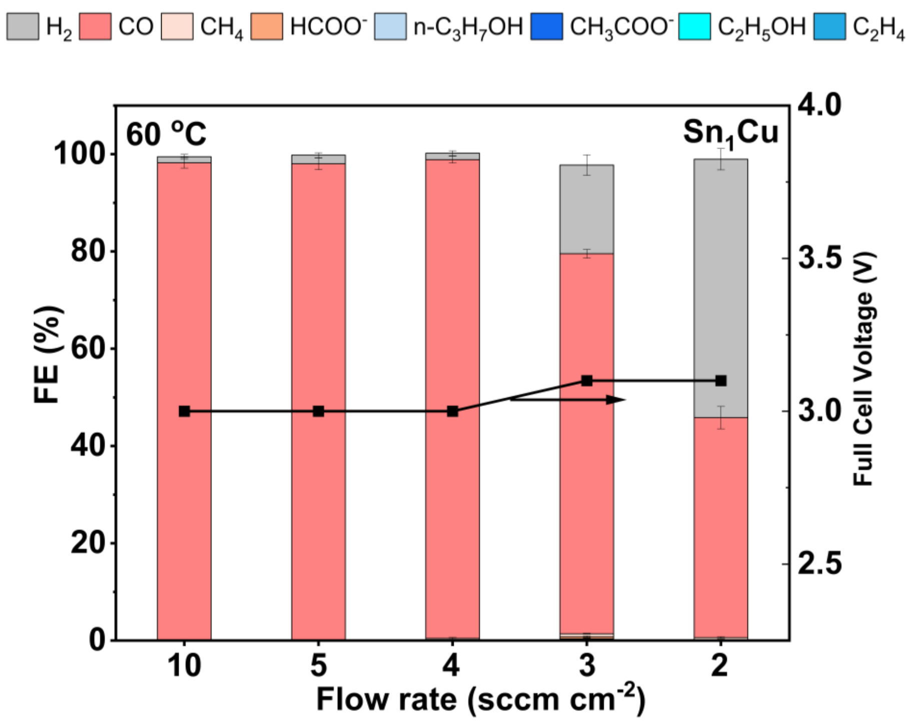

Figure S28. Product distribution of CO_2 reduction on Sn_1Cu at 300 mA cm^{-2} using different CO_2 flow rates.

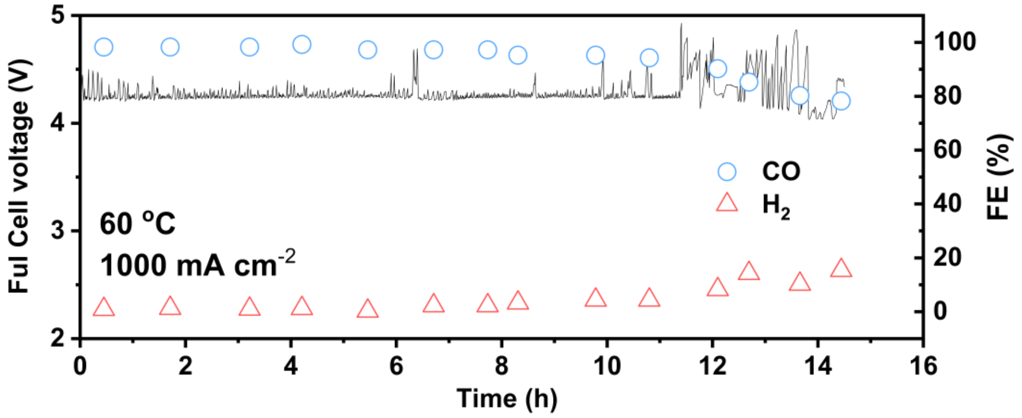

Figure S29. Stability of Sn_1Cu for CO_2 reduction at 1,000 mA cm^{-2} under 60 °C (40 μm AEM was employed for stability).

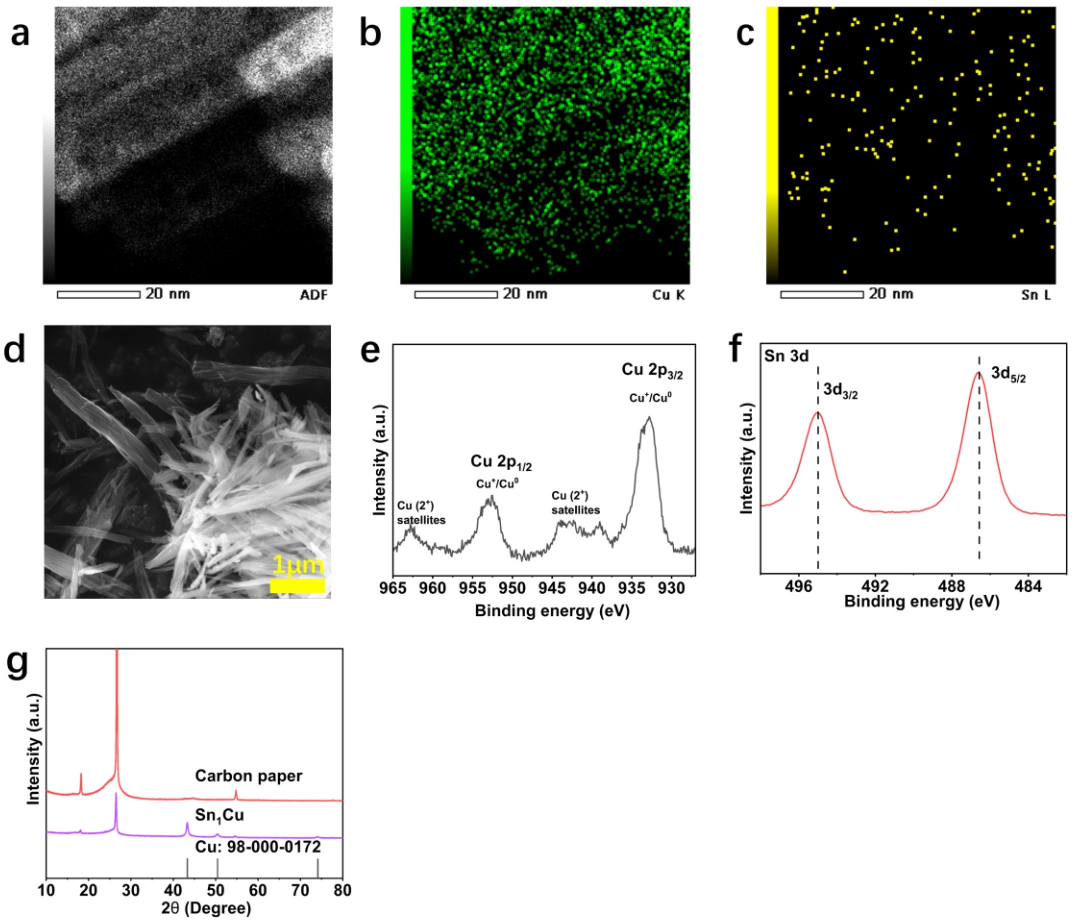

Figure S30. Structural and compositional characterization of Sn_1Cu after long-term stability testing (500 mA cm^{-2}, 95 h, 60 °C). (a) ADF-STEM image of Sn_1Cu. (b,c) Corresponding STEM-EDS elemental mappings of Cu (b) and Sn (c), demonstrating a homogeneous spatial distribution of Sn without detectable aggregation or phase segregation. (d) SEM image showing that the nanowire morphology is well preserved after electrolysis. (e) High-resolution Cu 2p XPS spectrum, where the attenuation of Cu^{2+} satellite features and the dominant Cu^0/Cu^+ signal indicate reduction of the surface under operating conditions. (f) High-resolution Sn 3d XPS spectrum confirming the presence of oxidized Sn species without evidence of metallic Sn clustering. The surface Sn content is determined to be 10.9 at%. (g) XRD pattern of the Sn_1Cu electrode after stability testing compared with the carbon paper substrate and reference Cu (PDF 98-000-0172), showing the absence of crystalline Sn or SnO_x phases and confirming the preservation of the Cu metallic structure.

Table S1. ICP-MS analysis of the electrolyte collected after stability tests at 500 mA cm^{-2} for 95 h.

Component	Result	Uncertainty	RL/MDC	units	Leaching percent
Cu	2.580E-002	±6.180E-003	3.960E-003	μg/mL	0.35±0.08%
Sn	<1.470E-002	<0.000E+000	<1.47E-002	μg/mL	below detection limit

Note: For ICP-MS analysis, 100 mL of recirculated electrolyte was sampled after the stability test. The corresponding Cu and Sn leaching percentages were calculated based on the measured concentrations, the total electrolyte volume, and a catalyst loading of 0.8 mg cm^{-2} on a 1 cm^2 electrode.

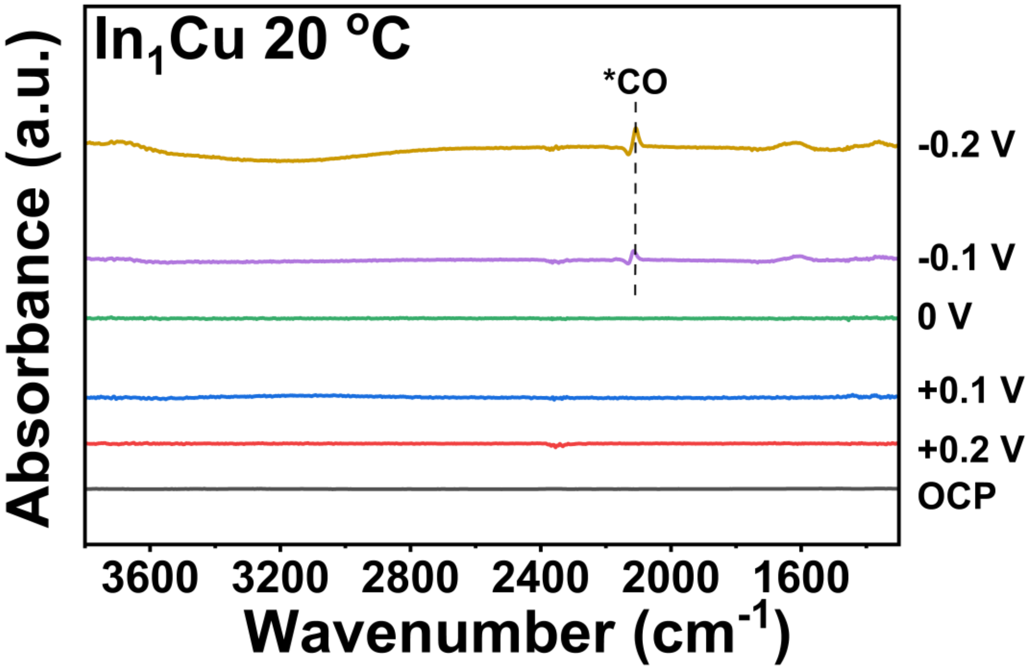

Figure S31. Low-overpotential in situ ATR-SEIRAS spectra of In_1Cu for determining the onset of CO formation. Spectra were collected at 20 °C from OCP to +0.2, +0.1, 0, -0.1, and -0.2 V vs RHE. The onset potential of spectroscopically detectable *CO is -0.1 V vs RHE, possibly due to positive shifts in the apparent equilibrium potential under low local CO partial pressure and cation effects.[S4-6]

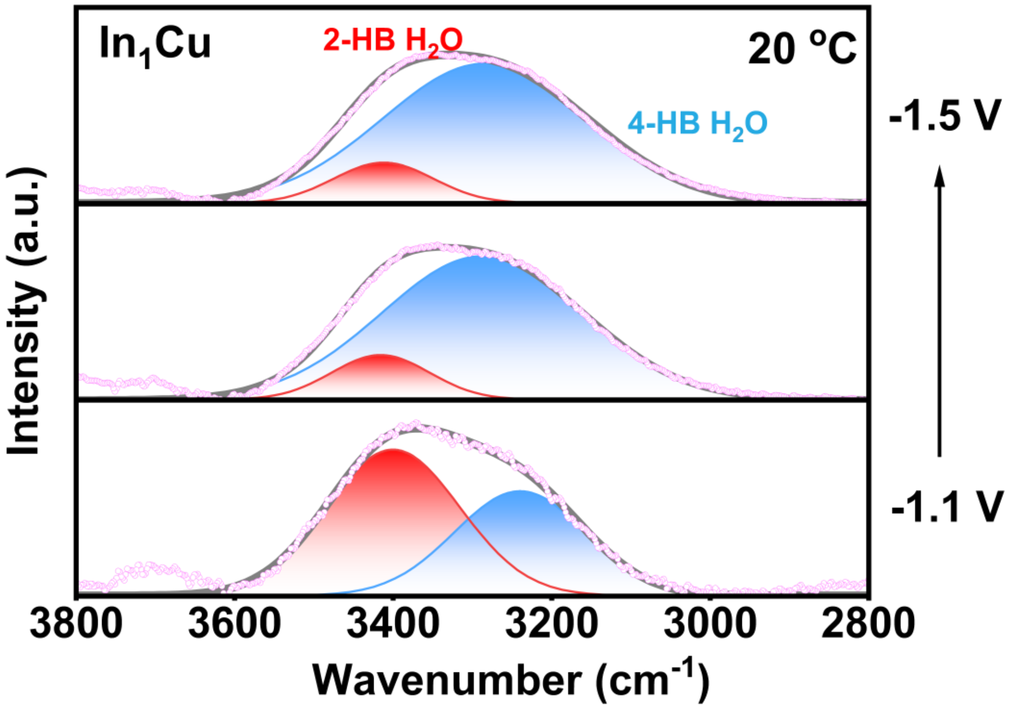

Figure S32. Gaussian deconvolution of the O-H stretching band of in situ ATR-SEIRAS of In_1Cu from -1.1 to -1.5 V vs RHE at 20 ºC.

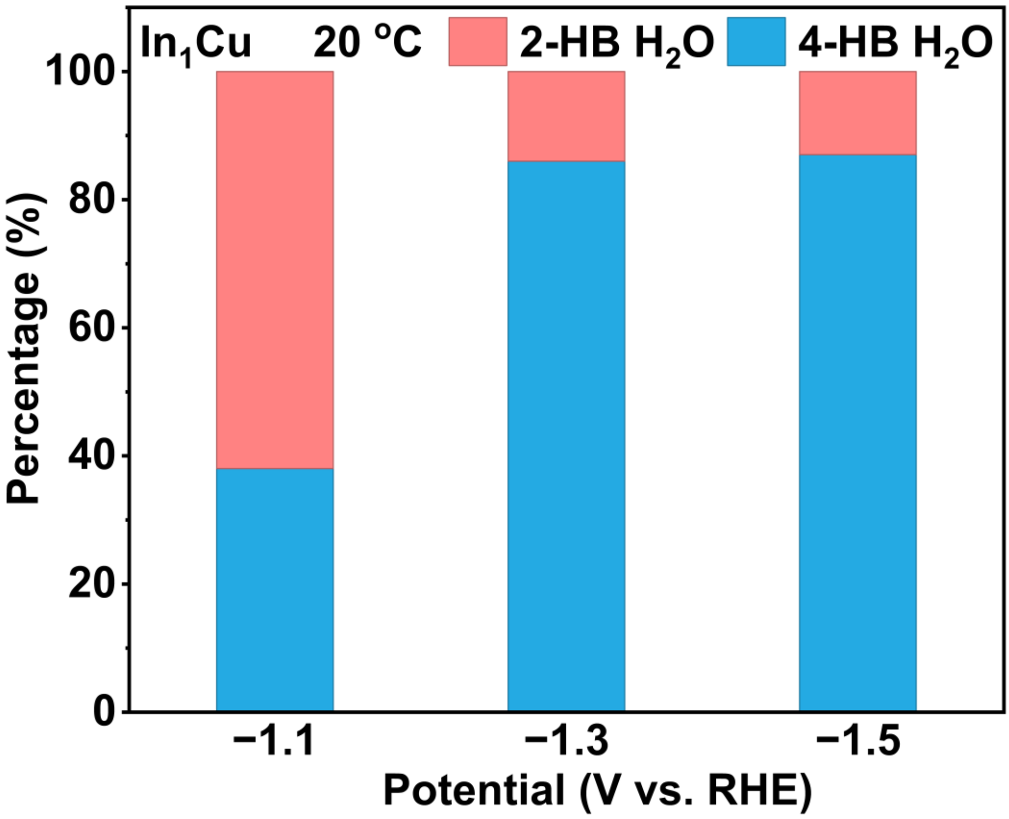

Figure S33. Percents of different interfacial water configurations of In_1Cu from -1.1 to -1.5 V vs RHE at 20 °C.

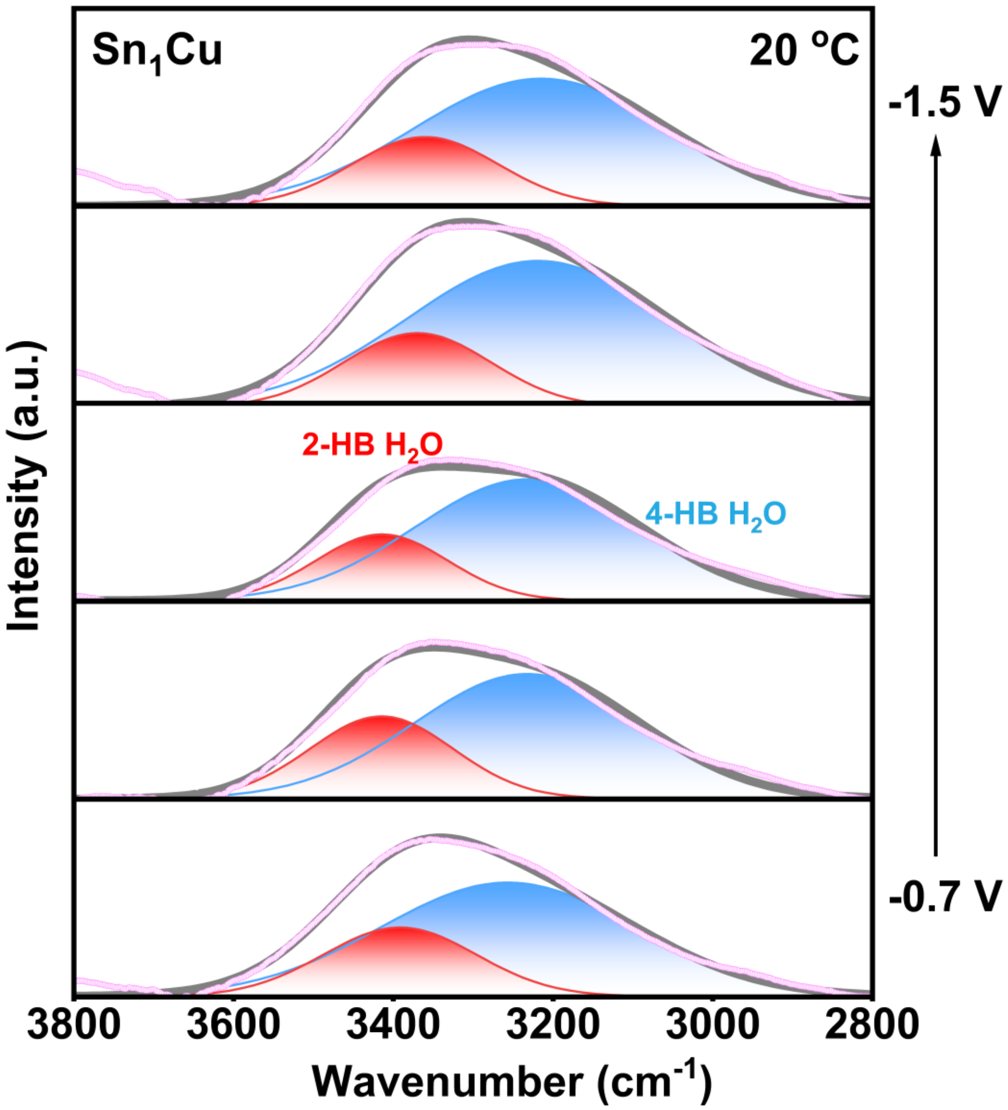

Figure S34. Gaussian deconvolution of the O-H stretching band of in situ ATR-SEIRAS of Sn_1Cu from -0.7 to -1.5 V vs RHE at 20 ºC.

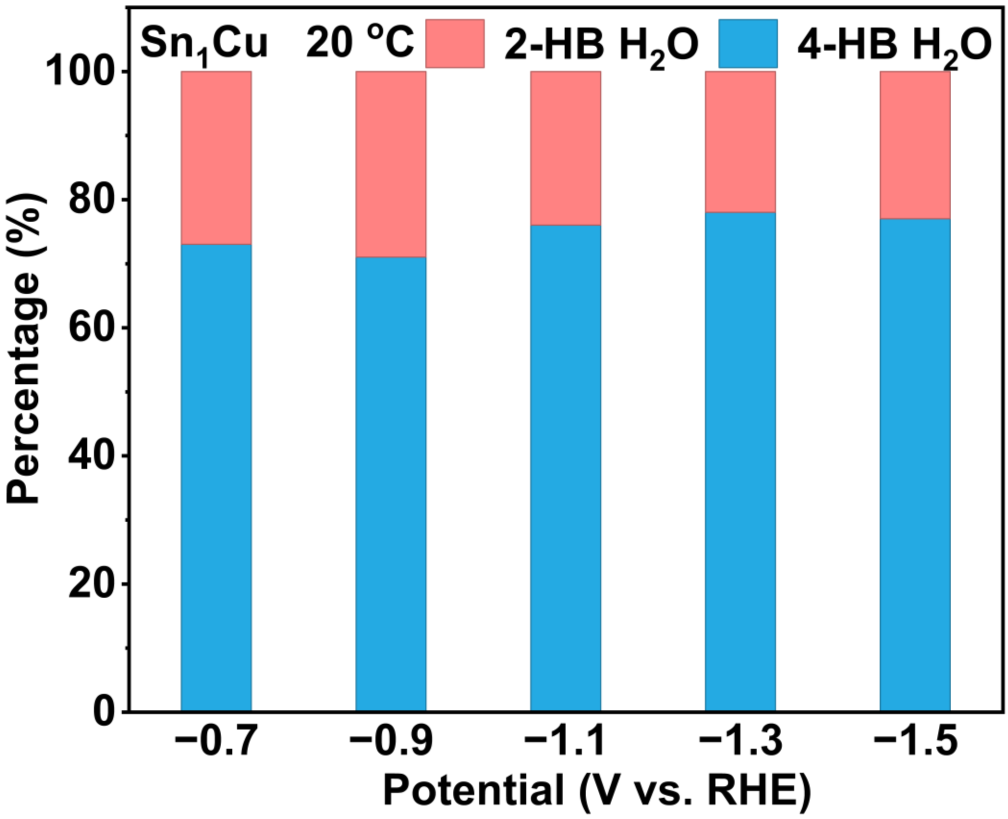

Figure S35. Percents of different interfacial water configurations of Sn_1Cu from -0.7 to -1.5 V vs RHE at 20 °C.

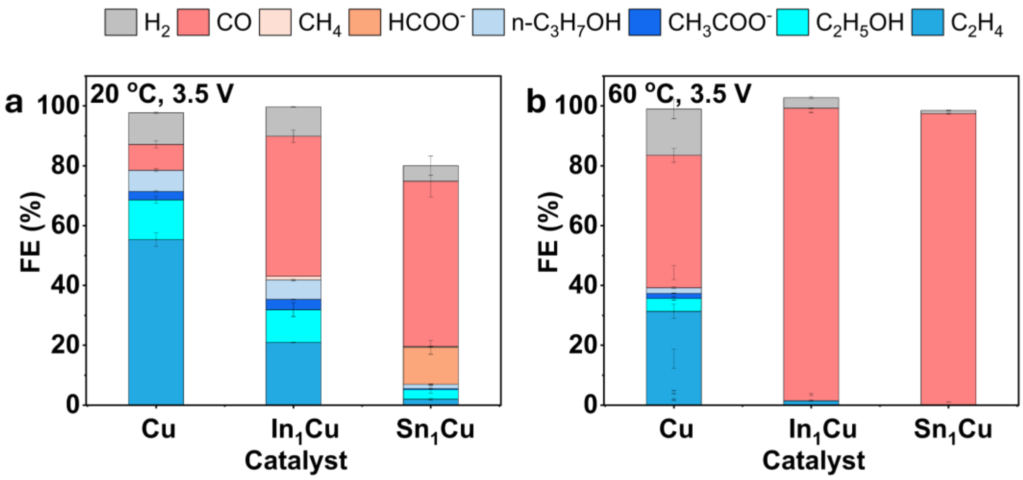

Figure S36. (a) Product distribution of CO_2 reduction on In_1Cu, Sn_1Cu, and Cu under 20 ^oC at a cell voltage of 3.5 V. (b) Product distribution of CO_2 reduction on In_1Cu, Sn_1Cu, and Cu under 60 ^oC at a cell voltage of 3.5 V. The FE values are means, while error bars represent the standard deviation from three independent measurements (n =3).

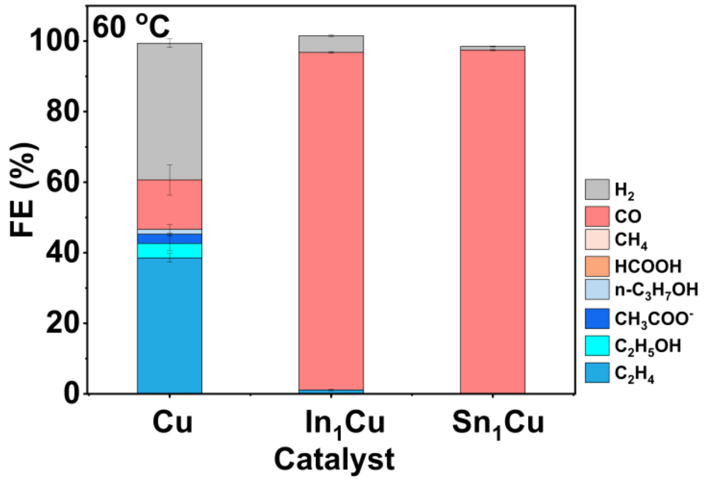

Figure S37. CO_2 reduction performance of Sn_1Cu, In_1Cu, and Cu at 60 °C and 1,100 mA cm^{-2}. The FE values are means, while error bars represent the standard deviation from three independent measurements (n =3).

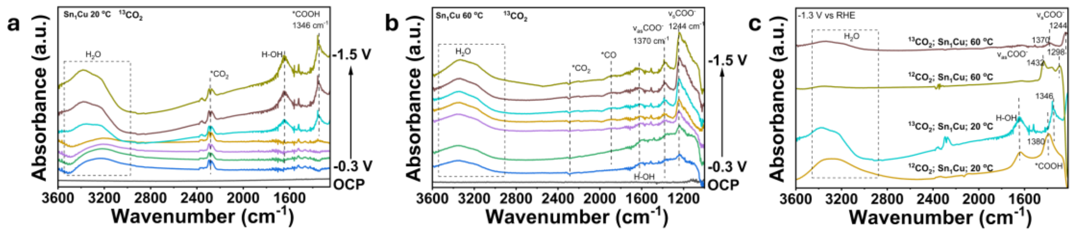

Figure S38. (a,b) In situ ATR-SEIRAS spectra of $^{13}CO_2$ reduction on Sn_1Cu at 20 °C (a) and 60 °C (b) from -0.3 to -1.5 V vs. RHE. (c) Comparison of in situ ATR-SEIRAS spectra of Sn_1Cu for $^{13}CO_2$ reduction and $^{12}CO_2$ reduction at 20 °C and 60 °C at -1.3 V vs RHE.

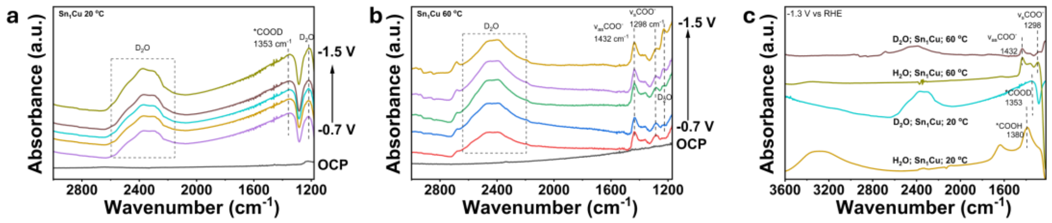

Figure S39. (a,b) In situ ATR-SEIRAS spectra of $^{12}CO_2$ reduction in solvent of D_2O on Sn_1Cu at 20 °C (a) and 60 °C (b) from -0.7 to -1.5 V vs. RHE. (c) Comparison of in situ ATR-SEIRAS spectra of Sn_1Cu for $^{12}CO_2$ reduction in solvent of H_2O and D_2O at 20 and 60 °C under -1.3 V vs RHE. The electrolyte is 0.1 M $KHCO_3$.

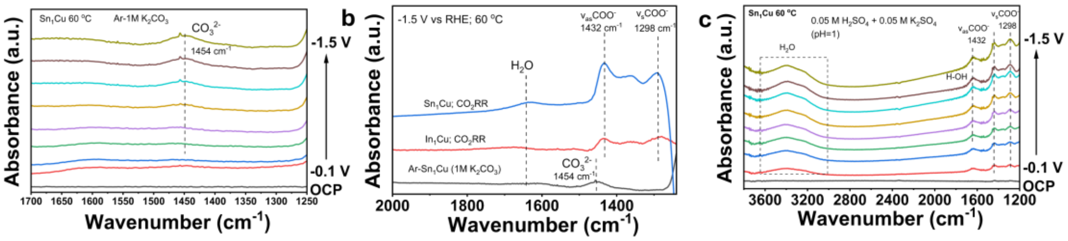

Figure S40. (a) In situ ATR-SEIRAS spectra of Sn_1Cu for Ar-purged 1 M K_2CO_3 reduction at 60 °C from -0.1 to -1.5 V vs. RHE. (b) Comparison of in situ ATR-SEIRAS spectra of carbonate and $^*COO^-$ from data in (a) and Figure 5a,b. (c) In situ ATR-SEIRAS spectra of Sn_1Cu for CO_2 reduction in acid electrolyte (0.05 M H_2SO_4 + 0.05 M K_2SO_4; pH = 1) at 60 °C from -0.1 to -1.5 V vs. RHE.

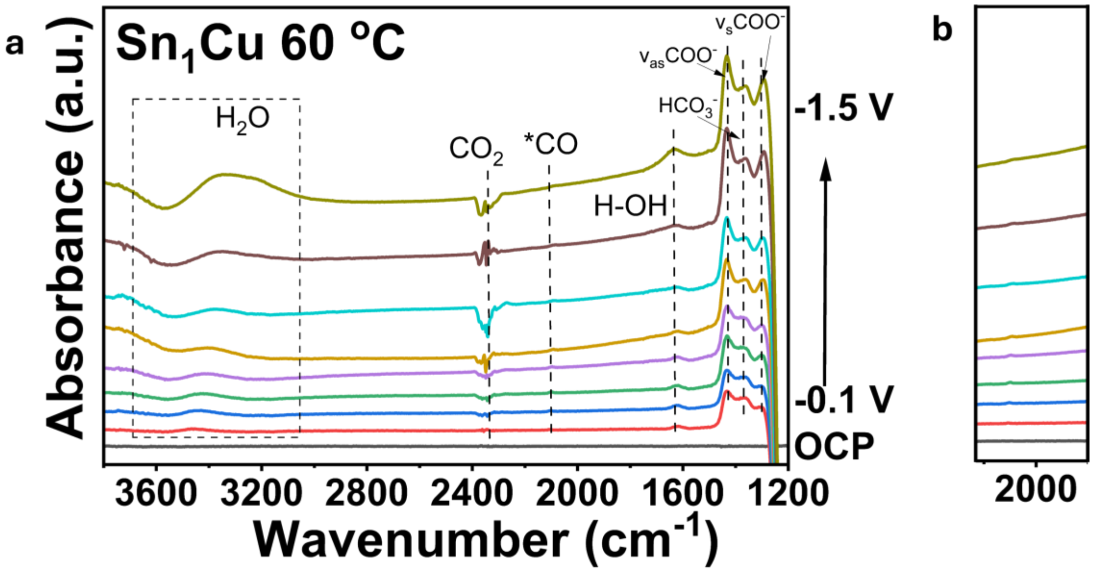

Figure S41. (a) In situ ATR-SEIRAS spectra of Sn_1Cu for CO_2 reduction at 60 ºC from -0.1 to -1.5 V vs. RHE (b) Enlarged view of *CO peak at 2100 cm^{-1} of (a).

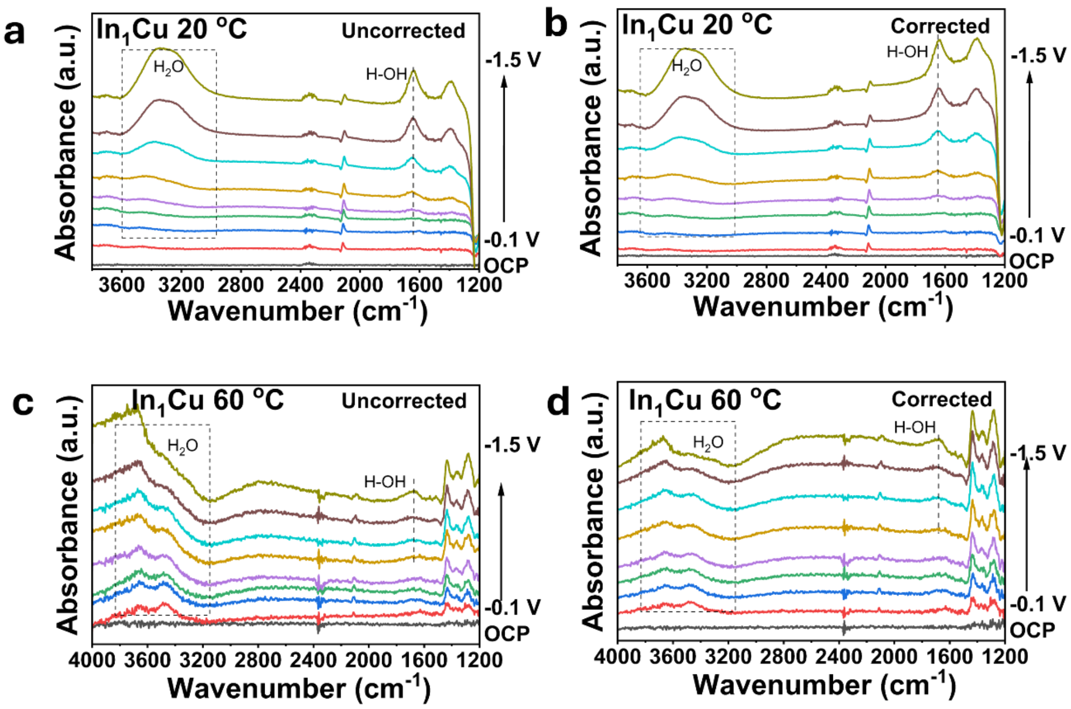

Figure S42. Comparison of uncorrected and baseline-corrected ATR-SEIRAS spectra of In_1Cu. (a,b) In situ ATR-SEIRAS spectra of In_1Cu recorded from -0.1 to -1.5 V vs RHE at 20 °C before (a) and after (b) baseline correction. (c,d) In situ ATR-SEIRAS spectra of In_1Cu recorded from -0.1 to -1.5 V vs RHE at 60 °C before (c) and after (d) baseline correction.

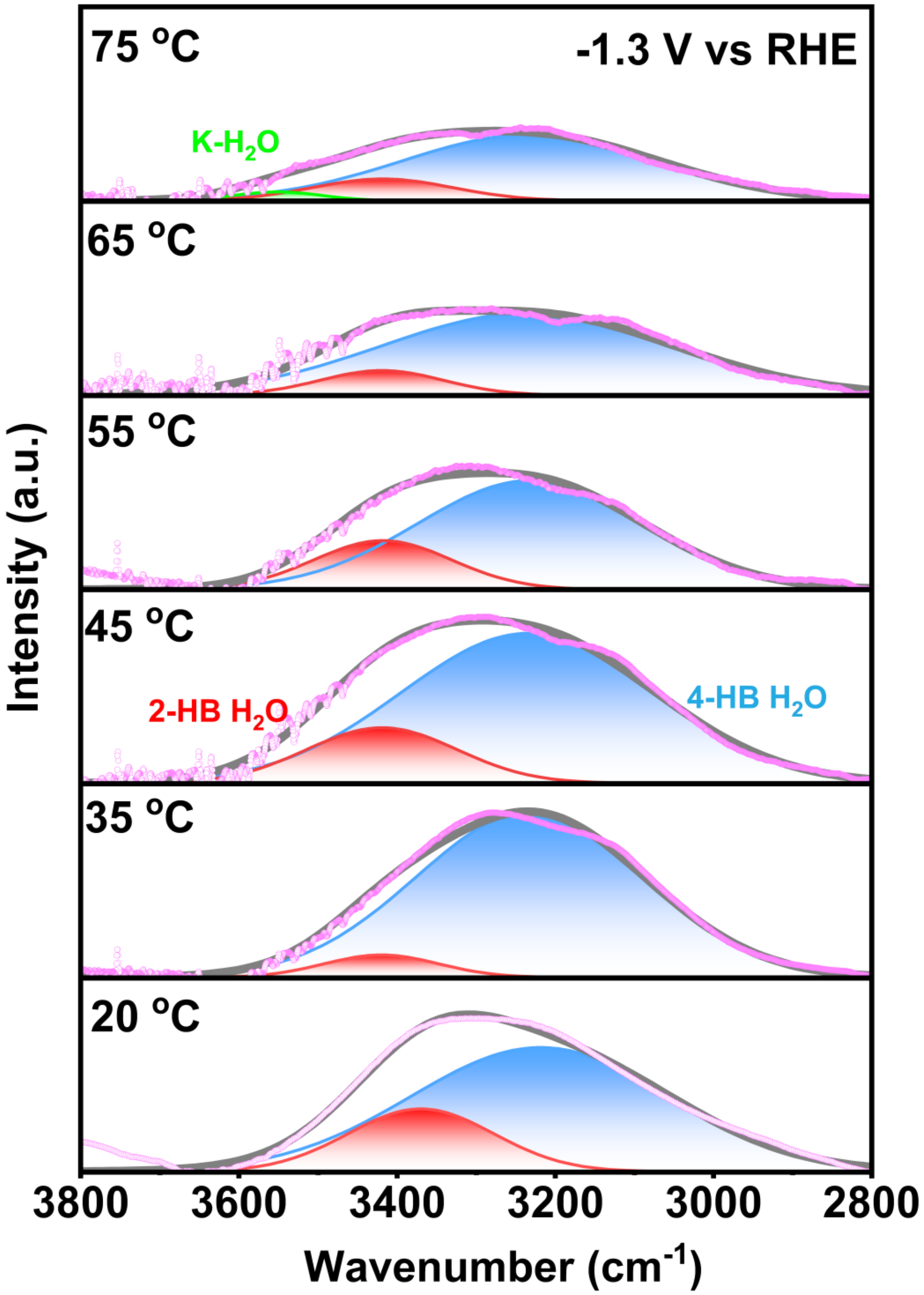

Figure S43. Gaussian deconvolution of the O-H stretching band of in situ ATR-SEIRAS of Sn_1Cu from 20 ºC to 75 ºC at -1.3 V vs RHE.

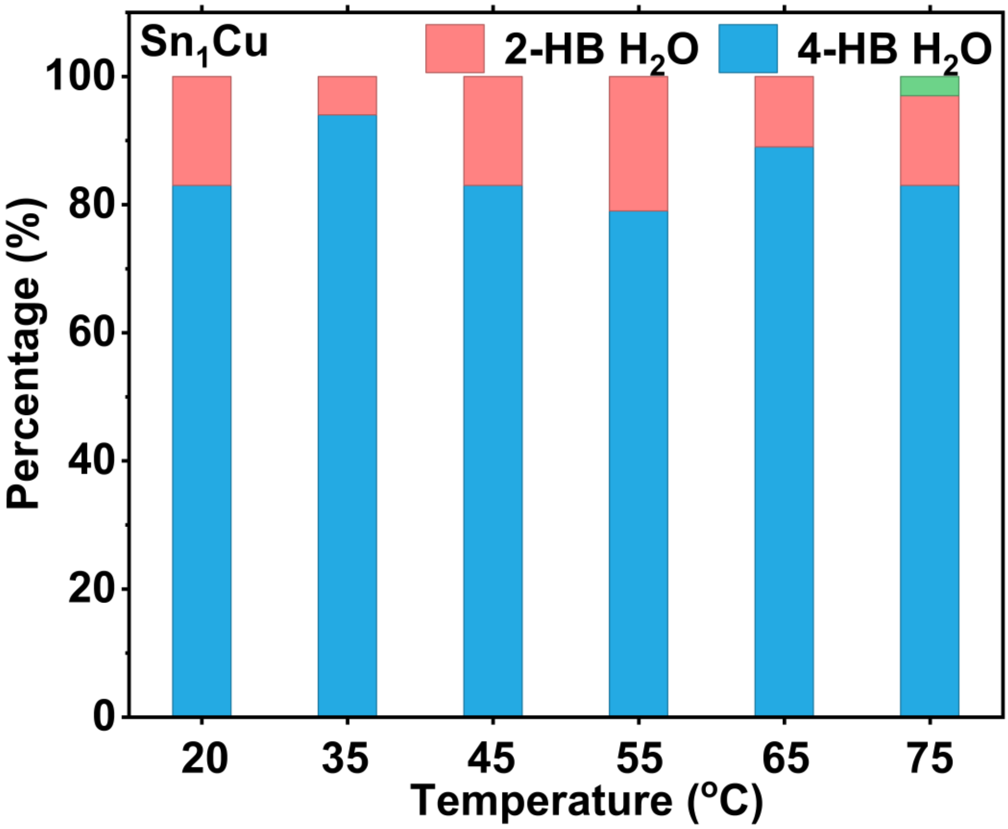

Figure S44. Percents of different interfacial water configurations of Sn_1Cu from 20 °C to 75 °C at -1.3 V vs RHE.

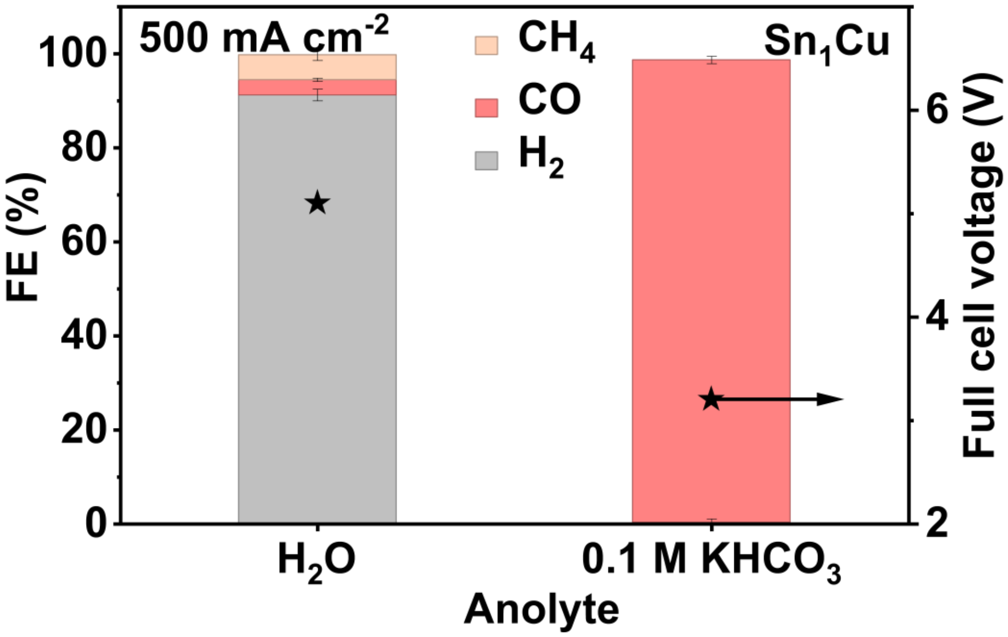

Figure S45. FE of all products of CO_2 reduction on Sn_1Cu at 60 °C using 0.1 M $KHCO_3$ anolyte and pure H_2O under 500 mA cm^{-2}.

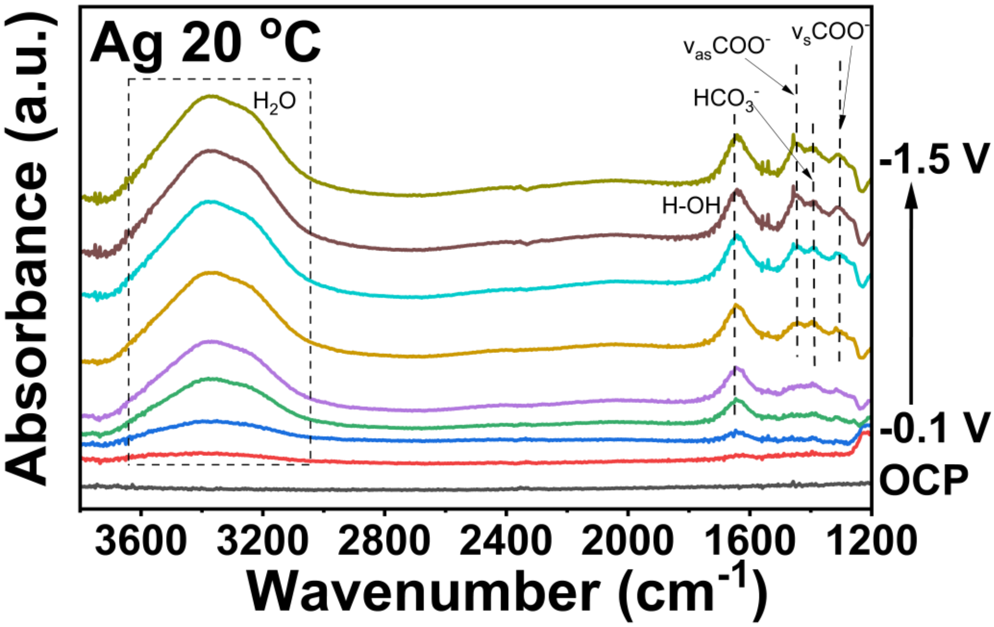

Figure S46. In situ ATR-SEIRAS spectra of Ag for CO_2 reduction from -0.1 to -1.5 V vs. RHE at 20 ^{o}C.

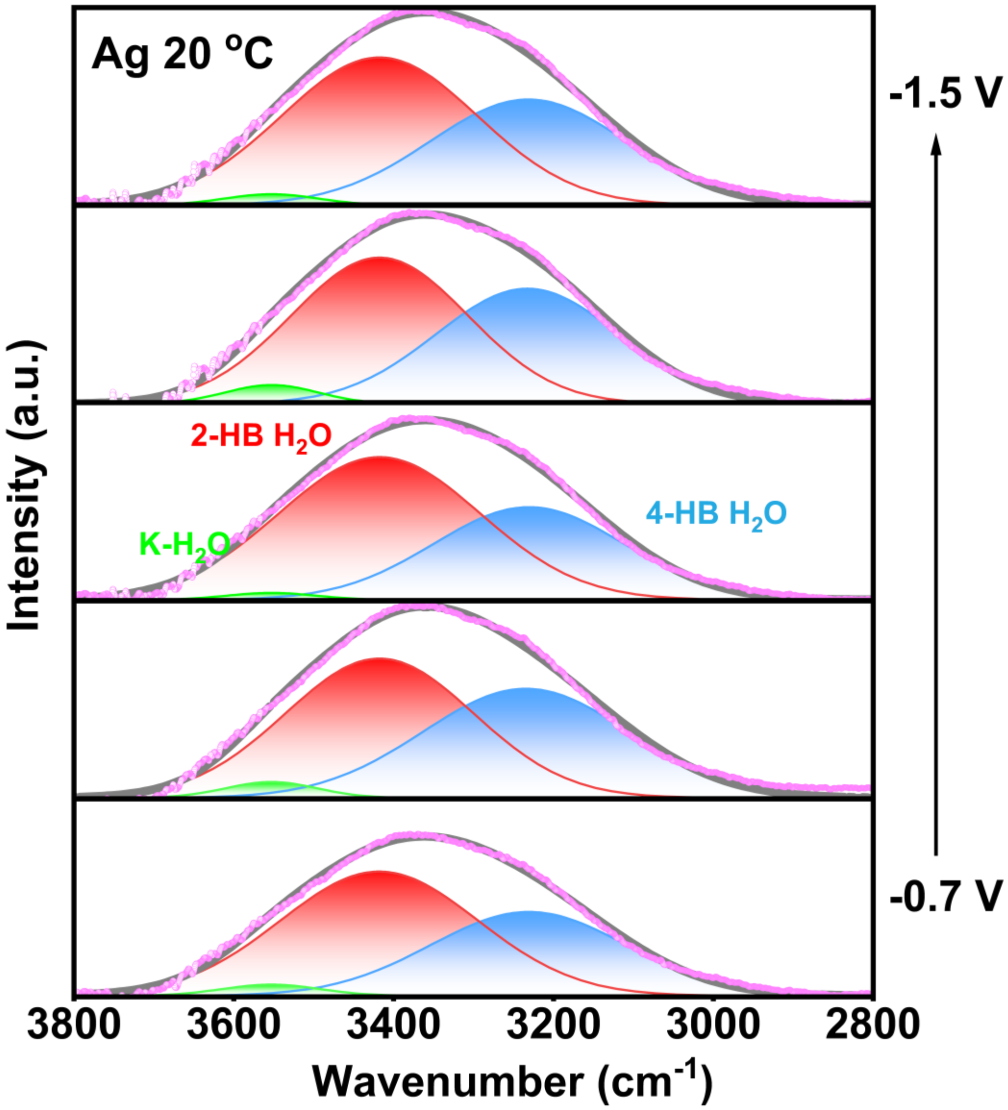

Figure S47. Gaussian deconvolution of the O-H stretching band of in situ ATR-SEIRAS of Ag from -0.7 to -1.5 V vs RHE at 20 ºC.

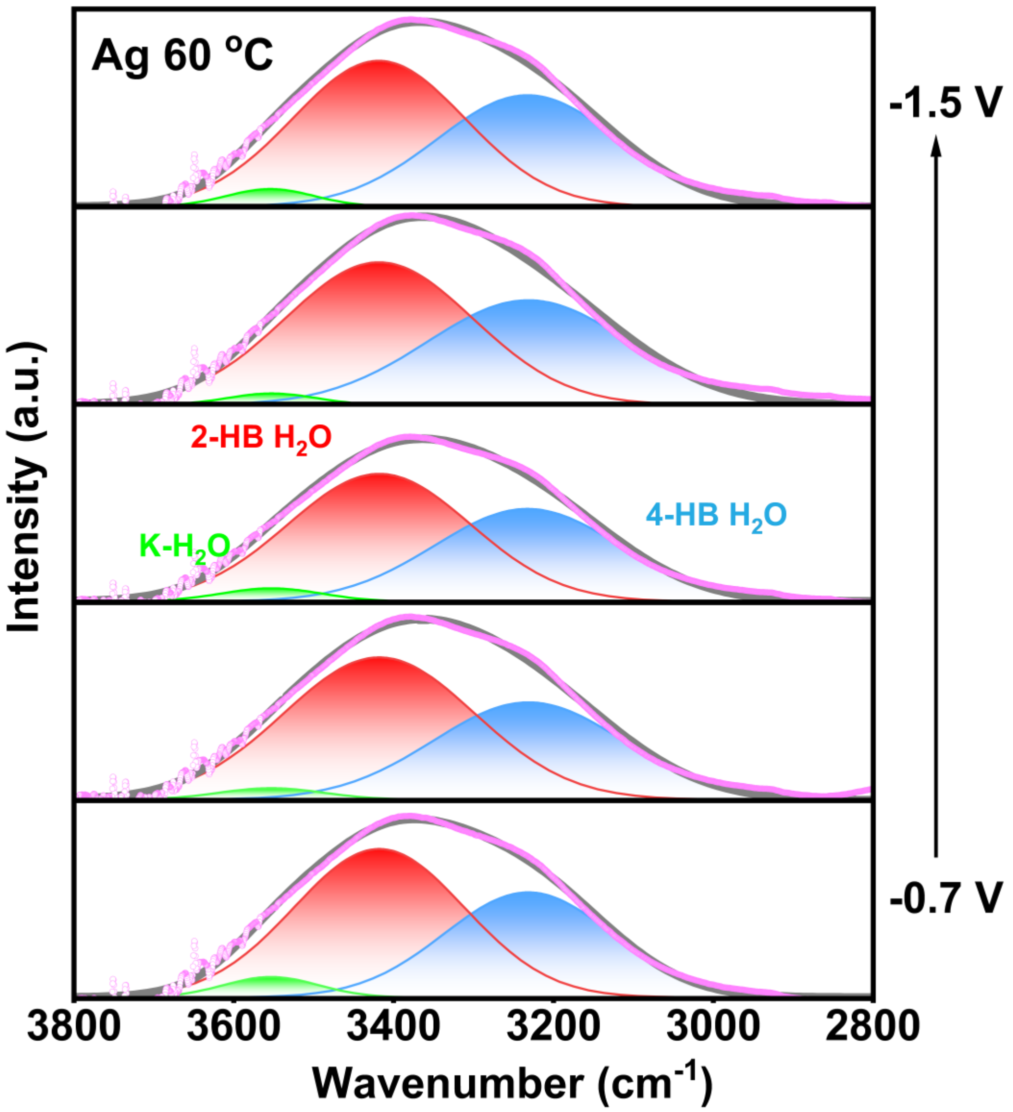

Figure S48. Gaussian deconvolution of the O-H stretching band of in situ ATR-SEIRAS of Ag from -0.7 to -1.5 V vs RHE at 60 ºC.

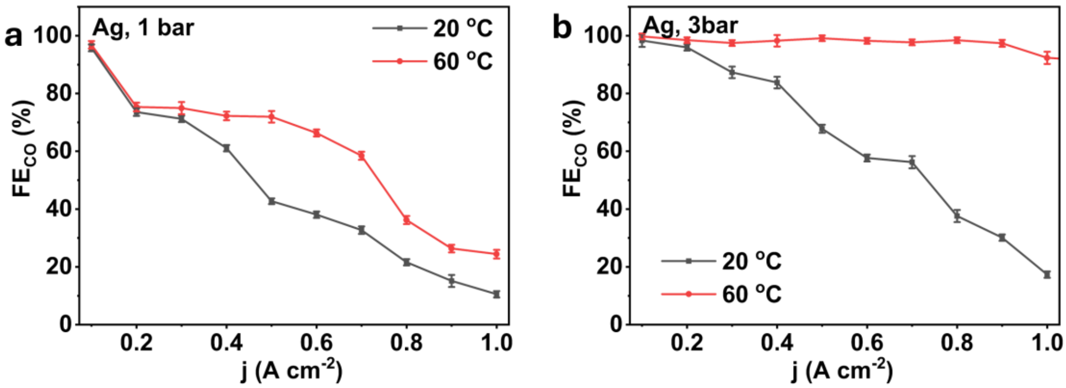

Figure S49. (a) FE of CO on Ag at both 20 °C and 60 °C under 1 bar CO_2 pressure. (b) FE of CO on Ag at both 20 °C and 60 °C under 3 bar CO_2 pressure. Tested in an MEA cell using 0.1 M $KHCO_3$ anolyte.

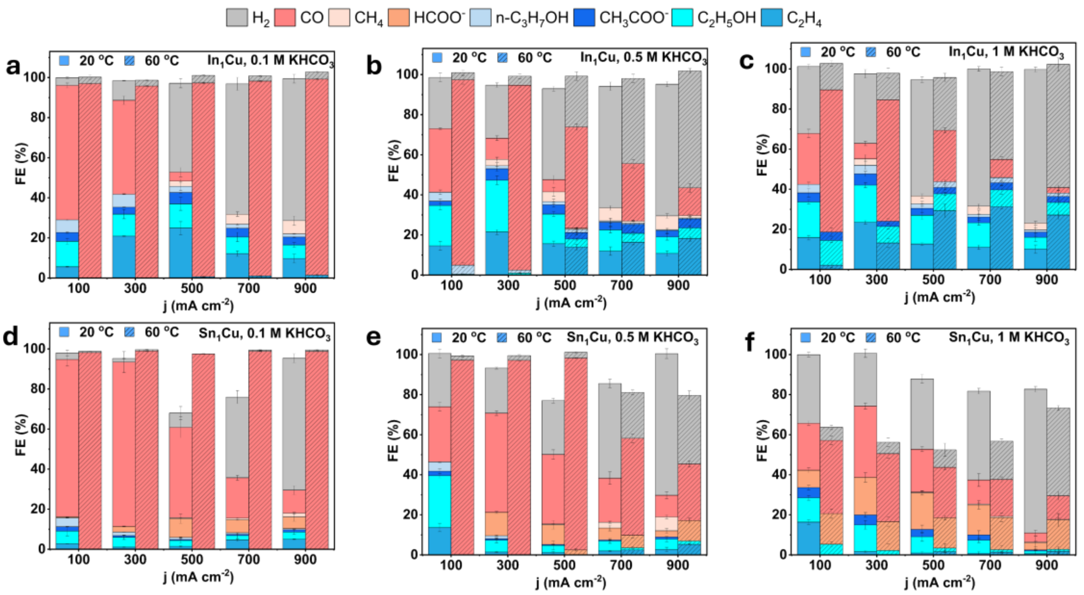

Figure S50. (a-c) CO_2 reduction performance of In_1Cu at 20 ºC and 60 ºC using 0.1 M $KHCO_3$ (a), 0.5 M $KHCO_3$ (b) and 1 M $KHCO_3$ (c). (d-f) CO_2 reduction performance of Sn_1Cu at 20 ºC and 60 ºC using 0.1 M $KHCO_3$ (d), 0.5 M $KHCO_3$ (e) and 1 M $KHCO_3$ (f). The FE values are means, while error bars represent the standard deviation from three independent measurements (n =3).

Supplementary references:

S1. Chen, X.; Kong, L.; Dong, D.; Yang, G.; Yu, L.; Chen, J.; Zhang, P. Synthesis and Characterization of Superhydrophobic Functionalized $Cu(OH)_2$ Nanotube Arrays on Copper Foil. *Appl. Surf. Sci.* **2009,** *255* (7), 4015-4019.

S2. Luo, W.; Xie, W.; Mutschler, R.; Oveisi, E.; De Gregorio, G. L.; Buonsanti, R.; Züttel, A. Selective and Stable Electroreduction of CO_2 to CO at the Copper–Indium Interface. *ACS Catal.* **2018,** *8* (7), 6571-6581.

S3. Simonson, H.; Klein, W. E.; Henckel, D.; Verma, S.; Neyerlin, K. C.; Smith, W. A. *Direct Measurement of Electrochemical Selectivity Gradients over a 25 cm^2 Copper Gas Diffusion Electrode. ACS Energy Lett.* **2023**, *8* (9), 3811-3819.

S4. Dunwell, M.; Lu, Q.; Heyes, J. M.; Rosen, J.; Chen, J. G.; Yan, Y.; Jiao, F.; Xu, B. The Central Role of Bicarbonate in the Electrochemical Reduction of Carbon Dioxide on Gold. *J. Am. Chem. Soc.* **2017**, *139*, 3774-3783.

S5. Han, Z.; Han, D.; Chen, Z.; Gao, J.; Jiang, G.; Wang, X.; Lyu, S.; Guo, Y.; Geng, C.; Yin, L.; Weng, Z.; Yang, Q.-H. Steering Surface Reconstruction of Copper with Electrolyte Additives for CO_2 Electroreduction. *Nat. Commun.* **2022**, *13*, 3158.

S6. Zhang, G.; Zhao, Z.-J.; Cheng, D.; Li, H.; Yu, J.; Wang, Q.; Gao, H.; Guo, J.; Wang, H.; Ozin, G. A.; Wang, T.; Gong, J. Efficient CO_2 Electroreduction on Facet-Selective Copper Films with High Conversion Rate. *Nat. Commun.* **2021**, *12*, 5745.
